# Genome-wide SNPs redefines species boundaries and conservation units in the freshwater mussel genus *Cyprogenia* of North America

**DOI:** 10.1038/s41598-021-90325-0

**Published:** 2021-05-24

**Authors:** Kyung Seok Kim, Kevin J. Roe

**Affiliations:** Department of Natural Resource Ecology and Management, Iowa State University, Ames, IA USA

**Keywords:** Conservation biology, Genetic variation, Speciation

## Abstract

Detailed information on species delineation and population genetic structure is a prerequisite for designing effective restoration and conservation strategies for imperiled organisms. Phylogenomic and population genomic analyses based on genome-wide double digest restriction-site associated DNA sequencing (ddRAD-Seq) data has identified three allopatric lineages in the North American freshwater mussel genus *Cyprogenia*. *Cyprogenia stegaria* is restricted to the Eastern Highlands and displays little genetic structuring within this region. However, two allopatric lineages of *C. aberti* in the Ozark and Ouachita highlands exhibit substantial levels (mean uncorrected *F*_ST_ = 0.368) of genetic differentiation and each warrants recognition as a distinct evolutionary lineage. Lineages of *Cyprogenia* in the Ouachita and Ozark highlands are further subdivided reflecting structuring at the level of river systems. Species tree inference and species delimitation in a Bayesian framework using single nucleotide polymorphisms (SNP) data supported results from phylogenetic analyses, and supports three species of *Cyprogenia* over the currently recognized two species. A comparison of SNPs generated from both destructively and non-destructively collected samples revealed no significant difference in the SNP error rate, quality and amount of ddRAD sequence reads, indicating that nondestructive or trace samples can be effectively utilized to generate SNP data for organisms for which destructive sampling is not permitted.

## Introduction

Precise information on the delineation of evolutionary lineages, their genetic composition and population sub-structure is imperative for guiding population restoration and the long-term preservation of biodiversity^1^. However, uncertainty about the accurate delimitation of species and the identification of distinct populations within species remain major challenges to the conservation of imperiled species that are also poorly studied from a taxonomic perspective^1–3^.

Freshwater mussels (Bivalvia: Unionidae), are one of the most endangered faunas in the world^4^. Estimates are that 65% of the North American mussel fauna are imperiled^5^, and additional extinctions (up to 50% of extant species) are projected over the next century^6^. Freshwater mussels are also important components of aquatic ecosystems and provide important ecosystem services in the systems they inhabit^7–9^. The life cycle of North American freshwater mussels includes a larval stage, called a glochidium, that must attach to a host fish to complete their development^10^. The larval dispersal distance, and therefore gene flow, of freshwater mussels is believed to depend largely on the dispersal capacity of their host fishes^11,12^. It has been shown that some freshwater mussel species utilize different host fishes in different river systems^13,14^, and research has shown that mussel larvae transform to juveniles at higher rates on sympatric populations of host-fish species^13,15^, indicating that the relationship between obligate parasitic mussel larvae and host fishes may be reinforced by co-evolutionary forces.

The freshwater mussel genus *Cyprogenia* inhabits high-gradient streams within the Mississippi faunal province which includes the Eastern, Ozark, and Ouachita highland regions of North America^16^. Currently, two species of *Cyprogenia* are recognized: the Fanshell *Cyprogenia stegaria* (Rafinesque 1820), which is listed as a federally endangered species (USFWS 1990) and is found east of the Mississippi River in tributaries of the Ohio River, and the Western Fanshell *Cyprogenia aberti* (Conrad 1850), which is found west of the Mississippi River in the Arkansas, White, Black, and Ouachita rivers^17,18^. Populations of *C. aberti* are declining and have been severely fragmented due to habitat loss and degradation by human disturbance^19^, and Harris et al.^18^ recommended that the status of *C. aberti* in Arkansas be changed from Threatened to Endangered. In many mussel species, the glochidia larvae are packaged, along with unfertilized eggs, into structures called conglutinates that resemble worms and facilitate host infection. The mature glochidia are almost completely transparent, and the color of the conglutinate lure results from the pigmentation of included unfertilized eggs^13,14^. In *Cyprogenia* species, the colors of conglutinates observed to date include brown, red, and white.

Previous phylogenetic and population genetic research on *Cyprogenia* have provided conflicting answers regarding the validity of the two nominal species. Phylogenetic research based on mitochondrial DNA (mtDNA)^20,21^ indicated that the two species of *Cyprogenia* were not reciprocally monophyletic, and that the divergent mitochondrial clades recovered in analyses were associated with the color of the conglutinate lures used by mussels to attract and infest host fishes^22^. In contrast, population genetic research using microsatellite markers^21,23^ supported the recognition of both *C. aberti* and *C. stegaria*, as allopatric species inhabiting the Western and Eastern highland regions respectively. Additionally, Chong et al.^23^ further advanced the hypothesis originally proposed by Serb & Barnhart^22^ that the mitochondrial lineages of *Cyprogenia* were associated with conglutinate color and these divergent mtDNA lineages were maintained by negative frequency-dependent selection by host fish. Despite the above findings, the taxonomic status of these species while clearer was still not fully resolved, and *C. aberti* has been assessed as Data Deficient due to these ongoing taxonomic issues (http://www.iucnredlist.org/details/6182/0).

Non-genomic markers are still the most commonly applied tool to address conservation genetic questions for freshwater mussels, however, the low information content and the potential non-neutrality of mtDNA markers, and costs and scoring issues associated with microsatellite markers have limited their usefulness. Recently, genomic data has been shown to be a valuable tool to address these challenges^24,25^. Application of population genomic tools, including reduced representation with restriction site associated markers allows for the harnessing of high-throughput next-generation sequencing to yield thousands of polymorphic genetic markers to address the conservation and management of this group. Genotyping-by-sequencing (GBS) or double digest restriction-site associated DNA sequencing (ddRAD) are reduced representation approaches which have generated highly informative single nucleotide polymorphisms (SNPs) for population genomic inference^24,26–29^ and phylogenomic research^25,27,30–41^ for numerous species across a broad range of taxa.

The availability of a large number of genome-wide SNP markers using the ddRAD-Seq approach has enabled high-density SNP discovery, genotyping, and genetic mapping at high resolution, and has provided solutions to unresolved questions concerning population genetic structure, species delineation, and to inform the management and conservation of a number of high-profile species of conservation concern^28,35,42^. Despite these advances, perceptions about the necessity of high quality starting material for the development of genomic libraries may have precluded the application of these techniques for endangered taxa because collecting permit restrictions only permit non-destructive sampling, which can yield small quantities of genomic DNA, are otherwise contaminated with non-target species or otherwise degraded^43^.

Here, we employed genome-wide ddRAD-Seq data to address species delimitation in *Cyprogenia*, document the genetic structuring, and attempt to further elucidate the relationship between conglutinate color and mtDNA lineages. We also explore and discuss possible barriers inhibiting gene flow and genetic connectivity across the range of *Cyprogenia*, the timing of population changes, as well as forces driving genetic divergence among mussel populations within the highlands. Finally, we used this opportunity to examine the utility of reduced representation approaches when only non-destructive samples are available as source material for genomic library creation by comparing the quality of results obtained from samples collected using a non-destructive method (cytology brushes) with those collected from mantle tissue biopsies.

## Results

STACKS parameter exploration identified a combination of assembly parameters (− m = 3, − M = 2, − n = 3, − max_locus_stacks = 3, with SNP model) as the best performing parameters (details in “Supplementary Information”), and this parameter setting was chosen for de novo ddRADseq assembly and SNP discovery, and for downstream genetic analyses. In total, 309,727 catalog loci were generated and 14,176 RAD loci passed sample/population constraints (− p 2, − r 0.60), where only loci that were present in 50% of four species-groups (*C. stegaria*_Eastern, *C. aberti*_Ozark, and *C. aberti*_Ouachita, and *Dromus dromas*) were retained. Total of 7,243 SNPs appeared to have a single SNP per locus with the mean depth of coverage of 7.24 (SD = 5.19).

### Genetic diversity of *Cyprogenia*

A total of 7243 SNPs were used to estimate population genetic parameters for four pre-defined mussel groups (*C. stegaria*_Eastern, *C. aberti*_Ozark, *C. aberti*_Ouachita, and *D. dromas*) as well as for ten rivers throughout the range of *Cyprogenia*. Across all three species, measures of genetic diversity were the highest in *C. aberti*_Ozark, followed, in decreasing order, by *C. stegaria*_Eastern, *C. aberti*_Ouachita and *D. dromas* (Table 1). Among rivers, genetic diversity was lowest in specimens from the Saline River and highest in the Black River (Table 1). Among highland regions, mean level of genetic diversity was the lowest in *C. aberti*_Ouachita, followed by *C. stegaria*_Eastern and *C. aberti*_Ozark. Genetic diversity estimates (Ho and He) were significantly different (*P* = 0.001; based on 999 permutation using OSx-statistic) among the three highlands regions.

**Table 1:** Measure of genetic Diversity and inbreeding coefficient for populations from rivers, Highlands and species.

Species/highland	River	Size	Na	Neff	Ho	He	*F*_IS_	*F*_ST_
*C. stegaria*/Eastern	Clinch	8	1.334	1.193	0.107	0.131	0.178	
	Green	10	1.409	1.221	0.121	0.144	0.162	
	Licking	24	1.462	1.225	0.120	0.141	0.153	
	Salt	9	1.393	1.215	0.122	0.142	0.142	
	All (Mean)	51	1.400	1.214	0.118	0.140	0.159	0.013
*C. aberti*/Ozark	Black	28	1.547	1.252	0.126	0.161	0.218	
	Spring	27	1.529	1.240	0.123	0.152	0.186	
	St. Francis	16	1.447	1.219	0.117	0.140	0.169	
	All (Mean)	71	1.508	1.237	0.122	0.151	0.191	0.073
*C. aberti*/Ouachita	Caddo	5	1.245	1.155	0.097	0.104	0.064	
	Ouachita	6	1.273	1.164	0.096	0.112	0.145	
	Saline	13	1.288	1.155	0.076	0.101	0.250	
	All (mean)	24	1.269	1.158	0.090	0.106	0.153	0.143
*D. dromas*	Clinch	8	1.131	1.076	0.031	0.051	0.393	

A total of 154 individuals except two geographically isolated individuals (Ozark_105 and Ozark_107) were included in this analysis.

*Na* mean number of alleles, *Neff* effective number of alleles (The number of alleles in a population, weighted for their frequencies), *Ho* observed heterozygosity, *He* expected heterozygosity assuming Hardy–Weinberg equilibrium, *F*_IS_ inbreeding coefficient, *F*_ST_ uncorrected for missing data.

Genetic differentiation (*F*_ST_) among rivers within each highland region was lowest in *C. aberti*_Ozark (*F*_ST_ = 0.0733), followed by *C. stegaria*_Eastern (*F*_ST_ = 0.013) and *C. aberti*_Ouachita (*F*_ST_ = 0.143) (Table 1). Pairwise genetic differentiation (*F*_ST_) among pairs of all ten rivers throughout the range of *Cyprogenia* showed similar patterns of differentiation for both raw and corrected data. Corrected *F*_ST_ values are lower than uncorrected values due to the use of averages to impute missing data (required for PCA analysis). The lowest and nonsignificant *F*_ST_ (*F*_ST_ = 0.007) was observed between the Licking and the Green rivers, and the highest (*F*_ST_ = 0.630, *P* = 0.001) was found between the Salt and the Saline rivers for the uncorrected data. Most pairwise comparisons were statistically significant after correction for multiple tests (Table 2). In general, high genetic differentiation was observed between the sampling locations of *C. stegaria* and *C. aberti*, but very high F_ST_ values were also observed between *C. aberti* from the Ouachita and Ozark highland regions.

**Table 2:** Pairwise *F*_ST_’s among 10 rivers throughout distribution range of genus *Cyprogenia* based on 7243 SNP loci.

Highland	River	*C. stegaria*_Eastern				*C. aberti*_Ozark			*C. aberti*_Ouachita		
		Clinch	Green	Licking	Salt	Black	Spring	St.Francis	Caddo	Ouachita	Saline
Eastern	Clinch	***–***	0.026	***0.026***	0.019	***0.197***	***0.259***	***0.244***	***0.271***	***0.248***	***0.215***
	Green	0.051	***–***	0.003	0.006	***0.241***	***0.304***	***0.287***	***0.307***	***0.286***	***0.248***
	Licking	***0.050***	0.007	***–***	0.005	***0.238***	***0.298***	***0.283***	***0.301***	***0.283***	***0.250***
	Salt	0.059	0.012	0.012	***–***	***0.212***	***0.275***	***0.257***	***0.278***	***0.259***	***0.226***
Ozark	Black	***0.506***	***0.508***	***0.513***	***0.508***	***–***	***0.018***	***0.030***	***0.134***	***0.119***	***0.098***
	Spring	***0.535***	***0.537***	***0.539***	***0.537***	***0.041***	***–***	***0.024***	***0.174***	***0.160***	***0.140***
	St.Francis	***0.563***	***0.558***	***0.554***	***0.561***	***0.106***	***0.073***	***–***	***0.173***	***0.155***	***0.136***
Ouachita	Caddo	***0.612***	***0.595***	***0.580***	***0.605***	***0.308***	***0.354***	***0.402***	***–***	0.007	0.036
	Ouachita	***0.609***	***0.594***	***0.582***	***0.604***	***0.311***	***0.357***	***0.400***	0.046	***–***	0.030
	Saline	***0.632***	***0.619***	***0.603***	***0.630***	***0.342***	***0.397***	***0.443***	***0.206***	***0.177***	***–***

*F*_ST_ values from raw data below diagonal and *F*_ST_ values from corrected data above diagonal.

A total of 146 individuals except two geographically isolated individuals (Ozark_105 and Ozark_107) were included in this analysis. Significance was tested by genic differentiation for each population pair from exact G test using default Markov chain parameters (Dememorisation: 10,000, Batches: 100, Iterations per batch: 5000). Bold Italicized indicates highly significant *F*_ST_ value. Corrected data refers to data that missing data were replaced with randomly drawn alleles based on the overall allele frequencies. Significance level was adjusted for multiple testing using Bonferroni correction.

Analysis of molecular variance (AMOVA) according to hierarchical groupings revealed a high degree of variance among the three highland regions, accounting for 21.6% of total variation (*F*_CT_ = 0.216, *P* = 0.001; using an Infinite Allele Model (IAM) and 999 permutations), which may largely reflect species divergence (Table 3). Significant but little genetic differentiation was observed among populations within regions (*F*_SC_ = 0.020, *P* = 0.001).

**Table 3:** Summary of hierarchical AMOVA analysis for *Cyprogenia* from the highland regions based on 7243 SNP loci.

Source of variation	%var	*F*-stat	*F*-value	c.i.2.5%	c.i.97.5%	P-value
**Three highlands**	(Ozark, Ouachita, and Eastern)					
Within individual	0.668	*F*_IT_	0.332	0.323	0.341	–
Among individual	0.100	*F*_IS_	0.131	0.125	0.136	0.001
Among population	0.016	*F*_SC_	0.020	0.019	0.022	0.001
Among highlands	0.216	*F*_CT_	0.216	0.209	0.223	0.001
Among population without grouping	–	*F*_ST_	0.179	0.174	0.185	0.001
**Two highlands**	(Ozark and Ouachita)					
Within individual	0.790	*F*_IT_	0.210	0.198	0.222	–
Among individual	0.100	*F*_IS_	0.113	0.104	0.121	0.001
Among population	0.027	*F*_SC_	0.030	0.027	0.033	0.001
Among highlands	0.082	*F*_CT_	0.082	0.074	0.091	0.107
Among population without grouping	–	*F*_ST_	0.069	0.064	0.075	0.001

A total of 146 individuals except two geographically isolated individuals (Ozark_105 and Ozark_107) were included in this analysis. AMOVA was conducted separately according to hierarchical groupings. Three Highlands correspond to the Ozark, Ouachita, and Eastern highland regions, consisting of two *Cyprogenia* species. Two Highlands correspond to Ozark and Ouachita Highlands, regions consisting of same species, *C. aberti*. Missing data are replaced with randomly drawn alleles based on the overall allele frequencies. Significance was tested using 999 permutations.

The estimated value for *F*_ST_ between mussels grouped by conglutinate color revealed low level of genetic differentiation (*F*_ST_ = 0.0377), indicating no evidence for population subdivision based on this phenotype. This difference is smaller than the values among rivers (Ozark: *F*_ST_ = 0.0733; Ouachita: *F*_ST_ = 0.143) within highland regions.

### Population structure of *Cyprogenia*

**Figure 1:**
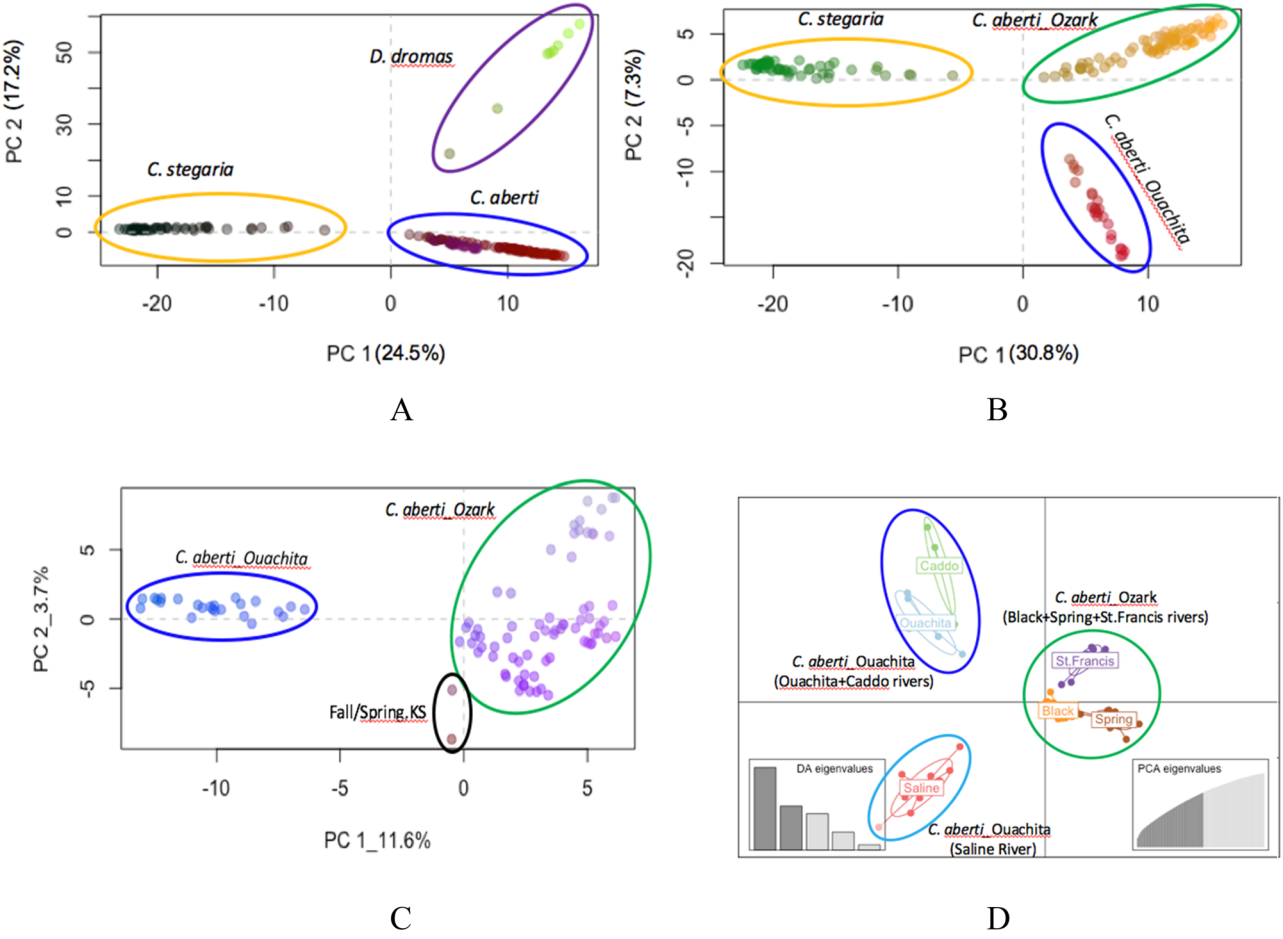
Results of the Principal Component Analyses (PCA) and Discriminant Analysis of Principal Components (DAPC) in R. In this Figure, genetic diversity is represented in two ways: by the distances (further away = more genetically divergent), and by the colors (more divergent colors = more genetically divergent). (**A**) PCA for entire 156 samples representing three freshwater mussel species (*Cyprogenia* + *Dromus*), (**B**) PCA for 148 samples representing genus *Cyprogenia*, (**C**) PCA for 97 samples representing *Cyprogenia aberti*. (**D**) DAPC for 97 samples representing *Cyprogenia aberti* (Number of PCA axes retained = 50, and number of PCA axes retained = 5).

A PCA along PC1 vs. PC2 for all 156 specimens showed a clear segregation of samples into three clusters (Fig. 1A). Further subdivision was observed along PC1 & PC3 and PC2 & PC3, in which *C. aberti* were further divided into two separate groups, *C. aberti*_Ouachita and *C. aberti*_Ozark. Specimens of *C. aberti*_Ozark were grouped with those of *D. dromas* for PC1 vs. PC3, whereas *C. aberti*_Ozark was grouped with *C. stegaria* for PC2 vs. PC3.

Additionally, a PCA excluding *D. dromas* samples displayed a clear separation into the three highland regions representing two *Cyprogenia* species (Fig. 1B). A PCA for 97 *C. aberti* specimens alone from Ozark and Ouachita highlands clearly distinguished specimens from the two highland regions, and specimens from the Fall and Spring rivers, KS (Fig. 1C). Finally, Discriminant Analysis of Principal Components (DAPC) for *C. aberti* specimens further subdivided *C. aberti* specimens into three discrete groups, 1: Caddo and Ouachita rivers in the Ouachita Highlands, 2: Saline River in the Ouachita Highlands, 3. Black, Spring, and St. Francis rivers in the Ozark Highlands (Fig. 1D).

**Figure 2:**
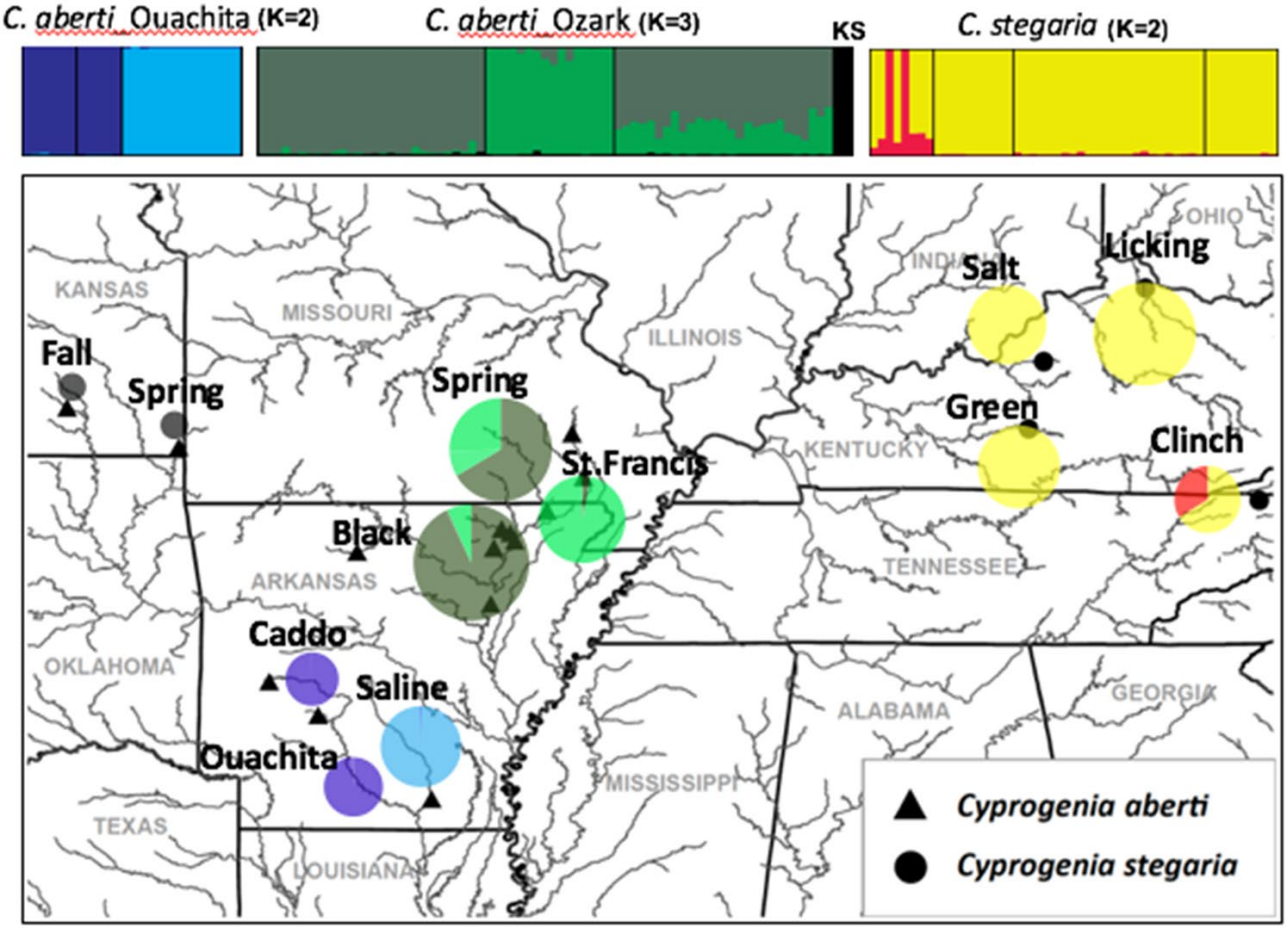
Geographic locations and pie charts of membership of each sampled population inferred by STRUCTURE analysis of *Cyprogenia*. The STRUCTURE plots show the posterior probability for individual assignments of samples to different genetic clusters based on the results of re-analysis of the original K = 3 clusters (Fig. S1). The optimal number of genetic clusters within Highlands was K = 2 for Ouachita, K = 3 for Ozark, and K = 2 for Eastern. Pie charts indicate proportions of membership of each sampled population to clusters inferred by STRUCTURE analysis for each Highland (see text for details). Circle size of pie charts is proportional to sample size. Sample sites for *Cyprogenia aberti* are marked as triangles and *Cyprogenia stegaria* as circles. Base map reproduced from Chong et al.^23^.

**Figure 3:**
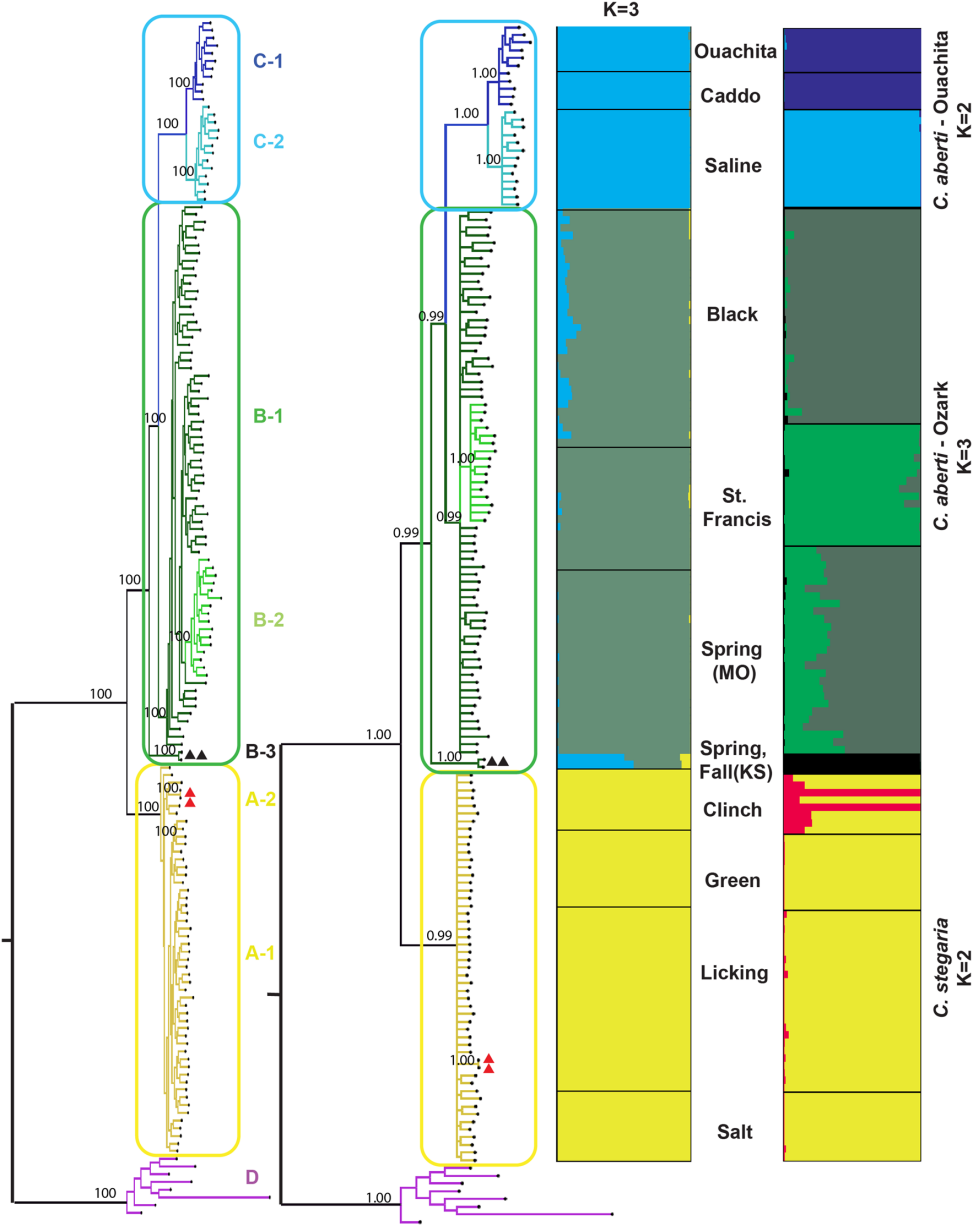
Phylogenetic relationships and population genetic composition of freshwater mussel species. Maximum likelihood (ML) tree (left) and Bayesian tree (right) were constructed based on 4395 informative sites from concatenated sequences of 9673 SNPs (see text for details). Bootstrap support values (ML) and posterior probability (Bayesian) respectively were provided at the node of major clade. The STRUCTURE plots show the posterior probability for individual assignments of samples to different genetic clusters. The main plot (left) shows the results for the optimal number of genetic clusters (K = 3) for 148 *Cyprogenia* specimens. The three smaller plots to the right show the results of re-analyses of samples from each Highland. The optimal number of genetic clusters was K = 2 for Ouachita, K = 3 for Ozark, and K = 2 for Eastern. Colors of clusters in the phylogenetic trees correspond to colors in the STRUCTURE plots and Fig. 2. Red triangles: Clinch river, TN (Eastern15 and Eastern18 in Table S1), Black triangles: Spring and Fall rivers, KS (Ozark105 and Ozark107 in Table S1).

STRUCTURE identified *K* = 3 as the optimal number of clusters for the *Cyprogenia* samples indicating little evidence of admixture among the three highland regions (Fig. 2 and left bar plot in Fig. 3). Further exploration of each cluster using STRUCTURE resulted in the identification of substructure within the Ouachita and Eastern highlands (*K* = 2), and the Ozark Highlands (*K* = 3). Based on these results, at least five genetically distinct clusters are identifiable within *C. aberti*, that correspond to the 1. Ouachita/Caddo rivers AR, 2. Saline River, AR (Ouachita Highlands), 3. Black/Spring rivers, AR, 4. St. Francis River, MO, and 5. Fall/Spring rivers, KS (Ozark Highlands) (right bar plot in Fig. 3). Interestingly, two specimens (Ozark105 and Ozark107) sampled from the Spring River and the Fall River in Kansas shared genetic composition indicative of both the Ozark and Ouachita Highlands (Fig. 3). Most of *C. stegaria* specimens in the Eastern Highland did not show any genetic structuring, but some specimens from the Clinch River had a genetic composition distinct from the other rivers (Fig. 3).

### Phylogenetic relationships among *Cyprogenia*

Maximum likelihood (ML) trees constructed using different filtering criteria produced nearly identical topologies, therefore for downstream analyses we used a data set in which SNPs were present in at least 60% of samples (Fig. S4). This same criterion was used for the Bayesian analysis. Both ML and Bayesian trees identified four distinct clades, *C. stegaria* (Clade A)*, C. aberti* (Clades B, C) and the outgroup, *D. dromas* (Clade D). The trees also indicated at least 6 subclades within *C. aberti*, all with strong bootstrap and posterior supports (A, B-1, B-2, B-3, C-1, C-2), reflecting river systems (Fig. 3).

Specimens from the Ouachita region were divided into two subclades, Clade C-1 (Caddo and Ouachita rivers, AR), and Clade C-2 (Saline River, AR). In the Ozark region, specimens from the St. Francis River, AR formed an internal clade within the Ozark clades. Notably, two individuals (Ozark 105 and Ozark 107), from the Spring and Fall rivers, in the Ozark region formed a clade (Clade B-3) sister to all other *C. aberti* clades*.* These individuals are also detected as a distinct cluster in the STRUCTURE analysis. *C. stegaria* specimens from the Eastern Highland region did not show clear pattern of geographic clustering (Fig. 3).

ML and Bayesian trees for *C. aberti* specimens with pre-defined conglutinate lure color displayed clear allopatric clades that corresponded to geographic regions (Ozark and Ouachita) and not with the color of the conglutinate lures (Fig. S5).

### Species tree and species delimitation of *Cyprogenia*

**Figure 4:**
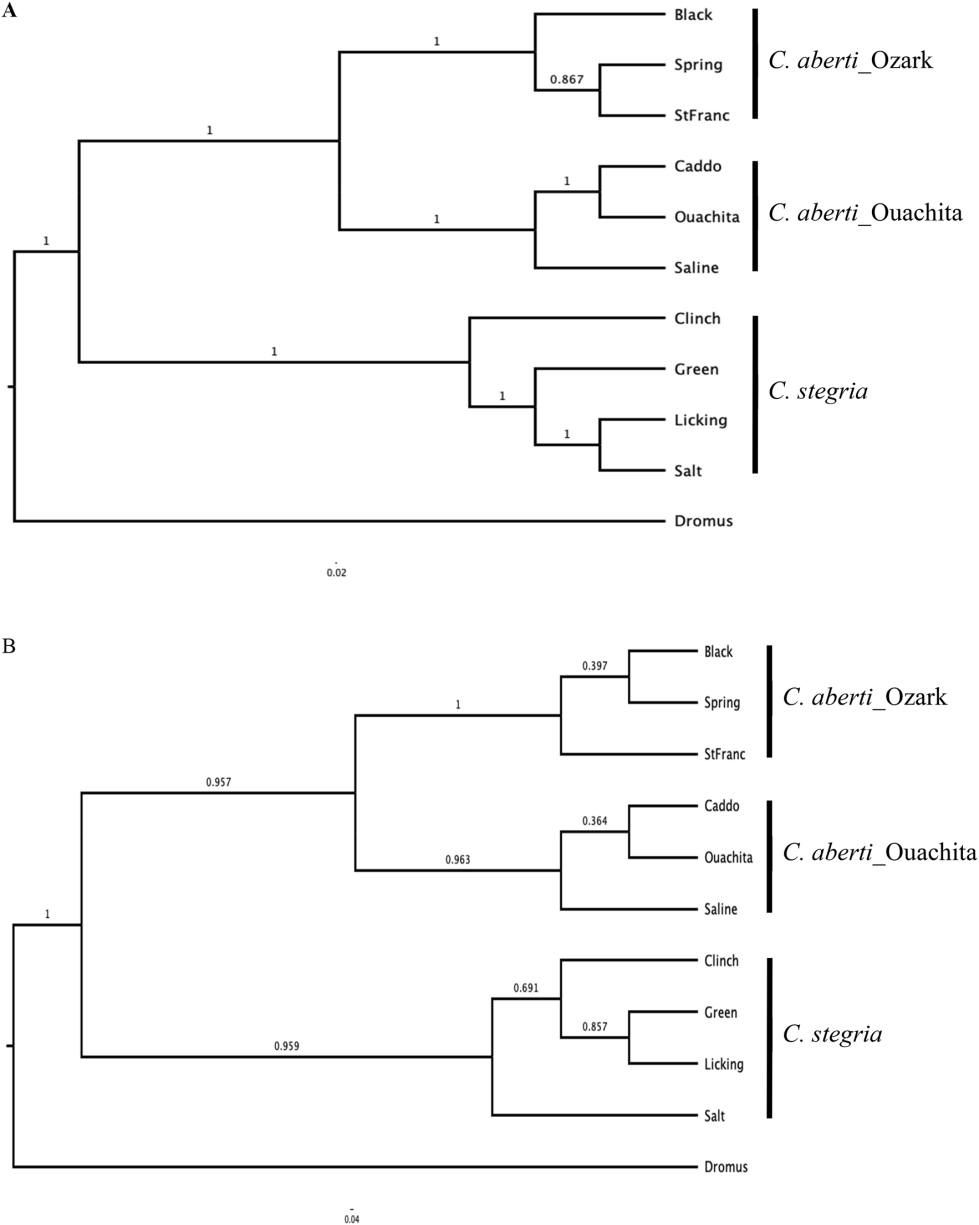
Species trees generated with Maximum clade credibility (MCC) and Median heights option in TreeAnnotator and with Transform branches (proportional) option in FigTree. Posterior probabilities are shown on the branch of each node. Total of 40 samples for 10 sampling sites (four samples per each sampling site) for *Cyprogenia* species were aligned and selected based on by selecting the individual samples with the highest coverage (smallest number of missing SNPs) (**A**) or by random sampling of individuals (**B**) using a custom Python script.

Species trees showed clear separation between *C. aberti* and *C. stegaria*. *Cyprogenia aberti* was further separated into two monophyletic groups reflecting highland regions (Fig. 4). Trees were generated based on two different sampling criteria, random sampling of individuals or by selecting the individual samples with the highest coverage (smallest number of missing SNPs). A tree selected based on coverage (Fig. 4A) revealed that, in the Ozark Highlands, the Black River was basal to the Spring and St. Francis rivers, and in the Ouachita Highlands, the Saline River diverged prior to Caddo and Ouachita rivers. These results indicated support for the results of the ML and Bayesian phylogenetic analysis.

The species delimitation results for the six *Cyprogenia* models are summarized in Table 4. The top-ranked model (Model2) included three species. In this model, *C. aberti* is divided into two species, Ozark Highlands (Black, St. Francis, Spring rivers) and Ouachita Highlands (Caddo, Ouachita, Saline rivers). Model 2 has the largest MLE value and received greater supported than the model (Model 1) that includes two *Cyprogenia* species, (current taxonomy model). Among models, the BF in support for Model 2 was decisive compared to other models (Table 4). The current taxonomy model (Model 1) was ranked 4th among models and was more supported than two paraphyletic models (Model 3 and Model 4). It is notable that a paraphyletic model (Model 3), where half the samples of the Ozark and Ouachita Highlands were intermixed with each other, was the least supported among models.

**Table 4:** Path sampling results for the six species delimitation models for *Cyprogenia* species shown in Fig. 2.

Model	Classification	No. species	MLE	Rank	BF	Detailed classification
1	Current taxonomy	2	− 55,853	4	–	Two *Cyprogenia* species: *C. aberti* and *C. stegaria*
2	Split *C. aberti*	3	− 53,201	1	− 5304	*C. aberti* was split into Ozark (Black, St. Francis, Spring) and Ouachita (Caddo, Ouachita, Saline)
3	Mix *C. aberti*	3	− 56,827	6	1949	Half of Ozark and Ouachita Highlands were intermixed
4	Reassign *C. aberti*	3	− 56,323	5	940	Black from Ozark was moved to Ouachita, and Saline from Ouachita was moved to Ozark
5	Reassign *C. aberti*	3	− 54,742	2	− 2222	Saline from Ouachita was moved to Ozark
6	Reassign *C. aberti*	3	− 55,285	3	− 1136	Black from Ozark was moved to Ouachita

All Bayes factor (BF) calculations are made against the current taxonomy model (Model 1). Therefore, positive BF values indicate support for current taxonomy model, and negative BF values indicate support for alternative model. The BF scale is as follows: 0 < BF < 2 is not worth more than a bare mention, 2 < BF < 6 is positive evidence, 6 < BF < 10 is strong support, and BF > 10 is decisive. Current and alternative species delimitation models were analyzed with the path sampling steps of 36, MCMC sample length of 100,000 for each path sampling step, alpha of 0.3, burnInPercentage = 10, and preBurnin of 10,000.

### Demographic changes in *Cyprogenia*

The past demographic changes of each of the sampling locations (five demographic models) were explored using Approximate Bayesian Computation (ABC) approach. The best-fit demographic model was selected by comparison of five models and the resultant estimations of current effective population sizes (*Ne*) for each sampling site are summarized in Table 5. All *Cyprogenia* populations showed either model 2 (DEC) or model 4 (INCDEC), or both models as the best fit model, depending on estimation method (direct or logistic regression) (Table 5), indicating a population decline after the Last Glacial Maximum (LGM), as supported by the best-fit demographic model (Table 5).

**Table 5:** Best fit demographic model and posterior distribution of the current effective population sizes (*Ne*) e and generation time (*T*) (Median and 95% credible interval) for each *Cyprogenia* sample site using ABC simulation implemented in DIYABC.

Highlands	Location/sample	Best scenario direct (logistic)	Parameter	Median	Quantile 5%	Quantile 95%
Ozark	Black	DEC (DEC)	*Ne*	4.70 × 10^2^	1.62 × 10^2^	9.73 × 10^2^
			*tt*	1.97 × 10^2^	1.13 × 10^2^	4.19 × 10^2^
	Spring	DEC (DEC)	*Ne*	7.78 × 10^2^	2.62 × 10^2^	1.60 × 10^3^
			*tt*	2.35 × 10^2^	1.19 × 10^2^	5.63 × 10^2^
	St. Francis	DEC (DEC)	*Ne*	2.00 × 10^3^	6.45 × 10^2^	3.93 × 10^3^
			*tt*	1.32 × 10^3^	3.16 × 10^2^	3.24 × 10^3^
Ouachita	Ouachita	DEC (DEC)	*Ne*	4.26 × 10^3^	9.88 × 10^2^	1.09 × 10^4^
			*tt*	2.33 × 10^3^	4.46 × 10^2^	4.26 × 10^3^
	Caddo	DEC (DEC)	*Ne*	3.79 × 10^3^	8.56 × 10^2^	1.07 × 10^4^
			*tt*	2.30 × 10^3^	4.45 × 10^2^	4.24 × 10^3^
	Saline	DEC (DEC)	*Ne*	3.29 × 10^3^	9.04 × 10^2^	7.31 × 10^3^
			*tt*	1.29 × 10^3^	2.39 × 10^2^	3.85 × 10^3^
Eastern	Salt	INCDEC (INCDEC)	*Ne*	1.55 × 10^3^	3.97 × 10^2^	4.26 × 10^3^
			*t1*	3.82 × 10^2^	1.30 × 10^2^	1.52 × 10^3^
			*t2*	3.62 × 10^3^	2.42 × 10^2^	4.42 × 10^3^
	Green	INCDEC (INCDEC)	*Ne*	3.78 × 10^2^	1.10 × 10^2^	1.29 × 10^3^
			*t1*	1.60 × 10^2^	1.06 × 10^2^	6.00 × 10^2^
			*t2*	3.90 × 10^3^	2.54 × 10^3^	4.46 × 10^3^
	Clinch	DEC (INCDEC)	*Ne*	2.35 × 10^3^	6.93 × 10^2^	5.16 × 10^3^
			*tt*	6.50 × 10^2^	1.63 × 10^2^	2.89 × 10^3^
	Licking	INCDEC (INCDEC)	*Ne*	2.23 × 10^2^	8.51 × 10	5.49 × 10^2^
			*t1*	1.22 × 10^2^	1.03 × 10^2^	2.06 × 10^2^
			*t2*	4.03 × 10^3^	2.62 × 10^3^	4.47 × 10^3^

Five demographic models proposed by by Cabrera and Palsbøll^81^ are evaluated. Model 1: CON (constant population size), Model 2: DEC (a single instantaneous decrease in population size), Model 3: INC (a single instantaneous increase in population size), Model 4: INCDEC (a single instantaneous increase followed by a single instantaneous decrease in population size), Model 5: DECINC (a single instantaneous decrease followed by a single instantaneous increase in population size). Time is in number of generations assuming a generation time of 5 years for *Cyprogenia*; *tt* denotes a unique event after the LGM, *t1* and *t2* denote an event after and before the LGM. Detailed prior model parameterization for five demographic models was provided in “Supplementary Information”. No mutation model parameterization was required for SNPs.

### Quality comparison between genetic materials using non-destructive and destructive methods

Genetic samples collected using cytology brushes showed a lower level of sample retention, compared to those from tissue samples. Forty six out of 88 nondestructive samples (52.3%) passed the quality filtering steps of the STACKS pipeline, whereas 110 out of 126 destructive samples (87.3%) passed the steps, which is statistically significant at the threshold of 0.05 (Fisher exact test statistic value = 0.022). However, after filtering, the amount (in estimates of mean ± SD) of ddRAD sequence reads (i.e. number in low quality, no radtags, retained and total loci) do not appear to differ between genetic samples using either method (Fig. S6). There was no significant difference in SNP error rates between duplicated genetic samples from nondestructive and destructive methods [SNP error rate: four duplicated cytology brush samples = 0.049 ± 0.014(SD) and four duplicated mantle biopsy samples = 0.056 ± 0.011(SD)]. Lastly, ddRAD sequences of randomly selected destructive (Fig. S7A: CSF_21, 3,023,365 loci) and non-destructive (cytology brush) (Fig. S7B: CSF_19, 3,881,449 loci) samples showed similar amount and pattern in the percentage of ddRAD sequences mapped to four genome sequences and PhiX sequences.

## Discussion

Understanding the population subdivision and genetic differentiation within a species can serve as a basis for defining units for conservation management^44^ and can inform management decisions to conserve species^45^. Detailed information on species delimitation, population genetic structure and genetic composition of populations at both regional and fine scales together will help establish the long-term management and design tailored conservation plans for freshwater mussel species that are among the most endangered faunas in North America. The present genome-wide SNP data provides improved resolution on species delimitation of *Cyprogenia* and also allowed detection of unrecognized population genetic structure at the regional scale.

*Cyprogenia* specimens were genetically more similar within regions than between different regions (Table 3). A biogeographic pattern reflecting highland regions has been also observed in other freshwater organisms, e.g. rainbow darter, rosyface shiner, streamline chub, etc., that are endemic to the highland regions of North America^46–48^. It has been proposed that Pleistocene glaciations have played an important role in determining faunal composition in the highlands of North America, in which a series of glacial cycles fragmented the region and its associated fauna^48–50^. Formation of the three major highland regions, the Ozark and Ouachita highlands west of the Mississippi River, and the Eastern Highlands east of the Mississippi River, from the contiguous Central Highlands of North America, during the Pleistocene has been discussed elsewhere^51^. Several case studies^47,52–55^ on population genetic structure for fish species in the North American Highlands evidenced diminished gene flow within and between these same highland regions. The results of the ABC simulations provide support for existing biogeographic scenarios within the Ozark and Ouachita regions. For example, the origin of the Ouachita River population via the admixture of the Caddo and Saline rivers is not inconsistent with hypotheses for the evolution of these drainages from their earlier pre-glacial condition^51,56^. Our scenario for the divergence of the St. Francis River also fits well with current hypotheses for the history of this river^51^, and although the divergence scenario for the Black and Spring rivers runs counter to our understanding of the origins of these rivers, it could accurately reflect the origin of the *Cyprogenia* populations that inhabit them.

Given the relationship between freshwater mussel larvae and host fishes, the reduced compatibility of freshwater mussels with allopatric populations of host fishes as compared to sympatric host fishes may serve to limit gene flow between mussel populations in different rivers^13,57^. Other factors such as anthropogenic disturbance (resource extraction, impoundments, etc.) and natural barriers can limit dispersal of host fishes within and between regions. These barriers may further reinforce genetic differences between host fish populations by inhibiting gene flow among Highlands, leading to population subdivision within and between highlands.

Phylogenetic trees derived from our genome-wide ddRAD-Seq SNP data identified three monophyletic groups in the genus *Cyprogenia*. *Cyprogenia stegaria* formed a distinct clade corresponding to the Eastern Highland Region, whereas *C. aberti* was divided into two major clades, which corresponded to the Ozark and Ouachita highlands of the Western Highland region (Fig. 2). Within the Ozark and Ouachita regions, *Cyprogenia* specimens were further subdivided into two distinct lineages delimited mainly by river systems. The three main genetic clusters in *Cyprogenia* identified by Bayesian model-based clustering analyses are consistent with the species delimitation analysis of *Cyprogenia* based on species trees in this study, and are in agreement with the results from the previous study using nuclear microsatellite markers^23^. Importantly, the present genome-wide ddRAD-Seq dataset also recognized additional genetically distinct clusters within the range of *C. aberti* not observed by Chong et al.^23^. These results indicate that the findings of host-fish compatibility trials, which indicated sympatric host fishes produced higher transformation rates of larvae to juvenile mussels than allopatric host fishes of the same species, may possibly have a genetic basis^22^.

Coalescent-based Bayesian species trees also indicated that *C. aberti* was comprised two clusters, Ozark and Ouachita, (Fig. 4). Species delimitation analysis revealed that a three species model was more supported than the two species model based on current taxonomy. Interestingly, the model, where samples of each highland region were intermixed (Model 3), showed the lowest support among the six competing species delimitation models (Table 4). Results based on species delimitation, phylogenetic analysis, population genetic structure, and PCA analyses, together support the recognition of three *Cyprogenia* species. However, more thorough and integrative understanding on geographical distribution, ecological and demographic characters, and host fishes use of the species, will help to address this issue.

Recognition of the three evolutionary lineages within *Cyprogenia* corresponding to the three highland regions supports continued recognition of *Cyprogenia stegaria* (Rafineque, 1820) as a distinct entity occurring east of the Mississippi River and occupying tributaries of the Ohio River basin. *Cyprogenia* that are found west of the Mississippi River, within the range of *Cyprogenia aberti* (Conrad, 1850) form two distinct lineages, one restricted to the Ozark region (White and St. Francis River systems) and the other in the Ouachita region (Ouachita and Saline river systems). Both of these entities are distinct enough to warrant recognition as separate species, however our current data set included only a small number of samples from the Spring and Fall rivers in Kansas, and we feel that additional samples from this area are needed to confirm this pattern. These samples formed a sister clade to the remaining western *C. aberti* populations and were also a distinct group in the Bayesian clustering analysis.

Since the Fall and Verdigris rivers are the type localities for the names *Unio aberti* Conrad, 1850 and *Unio popenoi* Call, 1855, the affinities of the Fall River populations and any extant populations in the Verdigris River are critical to the assignment to the name *Cyprogenia aberti* (Conrad, 1850). Because of the small number of samples from the Fall River and total lack of samples from the Verdigris River, additional taxonomic changes must await a more thorough geographic sampling of *Cyprogenia*.

Our Approximate Bayesian Computation (ABC) approach investigating demographic changes indicates that all *Cyprogenia* populations experienced a decline in size following the LGM (Table 5). Although some populations of *Cyprogenia* occur south of the maximal glacial extent, the impact of glaciation has been shown to still have impacts on population size and other demographic features in other taxa (e.g.^58^). The inferred reduction in population size and concomitant loss of genetic diversity likely contributed to the genetic differentiation revealed between these two highland regions in this study.

In this study, the combination of population genomic and phylogenomic findings suggests that conservation plans for *Cyprogenia* should consider at least 5, and possibly 7 distinct evolutionarily lineages when developing management plans; 1. *C. stegaria* in the Licking, Salt, and Green rivers, KY), 2. *C. “aberti”* in the Black/Spring rivers, AR, 3. *C. “aberti”* in the St. Francis River, MO, 4. *C. “aberti”* in the Ouachita/Caddo rivers, AR, 5. *C. “aberti”* in the Saline River, AR. These recommendations are justified by the fact that these five lineages have distinct genetic compositions and high level of genetic differentiation from other lineages (Table 2, Fig. 3). Individuals in these lineages form separate groups in PCA and DAPC (Fig. 1), and are further characterized by the lack of ecological exchangeability as evidenced by regional mussels’ compatibility to their local host fishes^13^. In addition to these, two additional regions, including *C. aberti* in the Spring and Fall rivers in Kansas and *C. stegaria* in the Clinch River in Tennessee warrant special attention as they appear to be genetically distinct entities, but have very limited geographic distributions.

The present ddRAD-Seq study employed genetic materials obtained by both nondestructive (cytology brush), and destructive (mantle biopsy) methods. Using cytology brushes (or swabs) is commonly used for extracting genomic DNA from endangered and threatened freshwater mussels. Despite the lower recovery rate of samples that passed the filtering steps of the STACKS pipeline in nondestructive genetic samples, our data showed that the quality and amount of ddRAD sequence reads were not significantly different between genetic samples using nondestructive and destructive methods. Our results therefore provide evidence that this nondestructive method can successfully be used for phylogenomic and population genomic studies using ddRAD-Seq analysis and can benefit conservation and management of endangered species such as freshwater mussels in North America.

## Materials and methods

### Mussel specimens and ddRAD-Seq library preparation

Genomic DNA for this study was derived from DNA extractions of specimens prepared for previous studies^20,21,23^. *Cyprogenia aberti* specimens were collected from rivers in Arkansas, Missouri, and Kansas, which occur within the Ouachita (Ouachita, Caddo, and Saline rivers) and the Ozark (Fall, Spring, St. Francis, Black, Current, Buffalo, and Strawberry rivers) highland regions (Fig. 2, Table S1). *C. stegaria* specimens were collected from the Eastern Highland Region (Licking, Salt, and Green rivers in Kentucky and the Clinch River in Tennessee).

Samples were collected nondestructively using cytology brushes and stored in the buffer supplied with the Puregene Buccal Cell Kit (Qiagen, Hilden, Germany) and DNA was extracted following the manufacturer’s instructions. Mantle biopsies were conducted by taking ~ 2 mm^2^ tissue and storing it in 95% ethanol (see Chong et al.^23^ for details). DNA was extracted from tissue samples using the DNeasy Blood & Tissue Kits (Qiagen, Hilden, Germany). For population genomic analyses, all *Cyprogenia* specimens were grouped according to their sampling locations, i.e., river. In addition, 14 *Dromus dromas* specimens were collected from the Clinch River, TN for this study and were used as an outgroup for phylogenetic analysis and for genetic comparison at the genus level. A total of 214 samples (200 *Cyprogenia sp.* + 14 *Dromus sp.*) were used in this study. Of these, 24 samples were randomly chosen as replicates to explore STACKS^59,60^ parameters for de novo assembly and SNP discovery (see below).

DNA libraries were prepared with modifications of Peterson et al.^61^. Briefly, a total of 200 ng of genomic DNA for each sample was normalized into 20 ng/μl, which was then digested using high fidelity restriction enzymes *Pst*1 and *Msp*1 (New England Biolabs). Resulting fragments were ligated to custom-made P1 containing sample-specific barcodes and forward primer annealing sites and P2 adapters with index and reverse primer annealing sites. Each individual was then PCR-amplified using Phusion Polymerase and following PCR conditions: 25 cycles at 98 °C for 20 s, 60 °C for 30 s and 72 °C for 40 s, with an initial denaturation step at 98 °C for 30 s and a final extension step at 72 °C for 12 min. PCR products were electrophoresed in 1.5% agarose gels and only samples with smeared PCR products were chosen for the next steps. Individually barcoded and indexed samples were then pooled into libraries and size-selected (325–500 bp) using a Blue Pippin (Sage Science) so that the remaining adapters and unbound primers/primer dimers can be removed. Each library was quantified and quality-checked using an Agilent BioAnalyzer 2100 (Agilent Technologies).

A total of 238 samples (214 samples + 24 replicates) were divided into 5 lanes (48 samples per lane, with one lane having 46 samples) and subjected to sequencing. Libraries were single-ended sequenced (100-bp) in separate flow cells on an Illumina HiSeq 3000 (Illumina Inc.) at the DNA Facility of Iowa State University. A simplified flowchart of our methods including ddRAD raw sequence reads, STACKS parameter selection, resultant datasets and data analyses is shown in Fig. S1.

### ddRAD locus assembly, STACKS parameter selection, and sample/loci filtering

The STACKS software pipeline, version 1.46^59,60^ was used for demultiplexing, quality filtering, de novo locus assembly, and SNP discovery (see “Supplementary Information”). We evaluated a variety of parameter settings in STACKS according to the recommendations of Mastretta-Yanes et al.^62^ to determine which combination balanced an acceptable SNP error rate and the number of loci retained (Details in “Supplementary Information”). A total of 156 specimens (148 *Cyprogenia* + 8 *Dromus*) were selected for phylogenetic and population genetic analyses. In this study, we applied the following STACKS population constraints to the 156 specimens. In STACKS, − p: refers to the minimum number of samples that a locus must be present in for inclusion of the locus in phylogenetic analyses: (1) for the phylogenetic study, only loci that were present in > 60% of all samples (i.e. 94 of 156) were retained, (2) for the population genetic study, only loci that were present in 50% of four species-groups (i.e. − p 2, here 2 of 4: *C. stegaria*_Eastern, *C. aberti*_Ozark, and *C. aberti*_Ouachita, and *Dromus dromas*), according to Chong et al.^23^, and in > 60% of all samples within each group (i.e. − r 0.6) were retained. Additionally, concatenated SNP data for all samples were also used for phylogenetic analysis of *C. aberti* specimens identified by conglutinate egg colors (14 red and 17 brown). Sample information for the 156 specimens used in this study is shown in Table S1. Raw ddRAD-seq data for each specimen are deposited into the NCBI SRA database (BioProject: PRJNA454895) under the accession numbers listed in Table S1.

### Population genetic diversity and structure

The STACKS populations program (− p 2, − r 0.6) was used to retain only those loci that were present in at least two of the four species groups (*C. stegaria*_Eastern, *C. aberti*_Ozark, *C. aberti*_Ouachita, and *D. dromas*) and present in over 60% of all samples within each group. In total, 14,890 RAD loci were obtained and 7243 SNPs (consisting of only the first SNP per ddRAD-Seq locus) were recovered and used for subsequent population genetic analyses.

Measures of genetic diversity for each of ten sampling locations (rivers) were calculated using GENODIVE^63^. Pairwise *F*_ST_ values to assess levels of genetic differentiation between locations were estimated following standard ANOVA^64^ using GENEPOP v.4.7^65^. Significance was tested through genic differentiation for each population pair using the exact G test with default Markov chain parameters (Dememorisation: 10,000, Batches: 100, Iterations per batch: 5000). Significance level was adjusted for multiple tests using Bonferroni correction.

To explore the effect of missing data in each locus, we compared pairwise *F*_ST_’s calculated for both raw data and corrected data in which missing data were replaced with randomly drawn alleles based on the overall allele frequencies using GENODIVE^63^.

GENODIVE was further used to conduct a hierarchical Analysis of Molecular Variance (AMOVA)^66^ to assess the partitioning of genetic variation among highlands, populations within highlands, individuals within populations and within individuals. Two separate AMOVA analyses were conducted (three highland regions vs. two highland regions). The comparison of three highland regions compared the Ozark, Ouachita, and Eastern highlands, which mainly accounts for variance between *Cyprogenia aberti* and *C. stegaria*. Whereas the comparison of two highland regions (Ozark vs Ouachita highlands), account for variance within *C. aberti*. Analyses were carried out using an Infinite Allele Model (IAM) and 999 permutations to assess its significance, where missing data were replaced by randomly drawn alleles based on overall allele frequencies.

We examined the distributions of individual multilocus genotypes in multivariate space, to assess the geographic structuring among individuals. We carried out a principal component analysis (PCA) based on 7243 SNP loci across all samples and *Cyprogenia* samples only using the adegenet package^67^ implemented in R^68^. A scatter diagram was plotted based on factor scores along the first two PC axes accounting for the most variation. We also conducted a Discriminant Analysis of Principal Components (DAPC) using an interactive web interface for DAPC distributed with the package [using R command: adegenetServer(what = "DAPC")]. DAPC aims to provide an efficient description of genetic clusters using a few synthetic variables that are constructed as linear combinations of the original variables (alleles) which have the largest between-group variance and the smallest within-group variance^69^. We used the default parameters except selecting the option of suggested number of PCA components.

To infer the most likely number of genetic clusters, we applied a Bayesian clustering approach implemented in STRUCTURE 2.3.4^70^. The admixture ancestry model was used with correlated allele frequencies and no prior on population membership. The initial analysis of the complete dataset was conducted ten times for each K value (the number of inferred genetic clusters) from 1 to 10 with 200,000 Markov Chain Monte Carlo (MCMC) generations and a burn-in of 100,000. The optimal K value was inferred from ad hoc posterior probability models of [Pr(X|K)]^70^ and deltaK^71^ using STRUCTURE HARVESTER^72^. To assess the presence of substructure within major clusters, subsequent analyses were conducted within each of main clusters, using the same parameters as described above. A STRUCTURE bar plot to infer the genetic composition of individuals was visualized using DISTRUCT v.1.1^73^.

### Phylogenetic analyses

To explore the effect of missing data, we compared data sets generated by four different filtering criteria using the STACKS population program. We varied this criterion in order of strictness (i.e., inclusion of loci shared by fewer to more samples); − p 78, − p 94, − p 109, and − p 125, which only retains loci present in at least 50%, 60%, 70% and 80% of the 156 samples respectively. Using the STACKS population program (–phylip_var) and the *D. dromas* specimens as an outgroup, these four data sets of concatenated SNPs were used to investigate evolutionary relationships among the156 specimens. These four different filtering criteria respectively generated concatenated SNPs of 15,829, 9673, 4674, and 1283, each with parsimony-informative sites of 7440, 4395, 2014, and 511. Phylogenetic trees were constructed with Maximum likelihood (ML) analyses in IQtree 2.1.2^74,75^ with 1000 ultrafast bootstrap replicates (− bb), 10,000 iterations (− nm) and Ascertainment bias correction (+ ASC) model. The TVM + F + ASC + G4 model of sequence evolution was selected as a best-fit model according to a Bayesian Information Criterion (BIC) model selection implemented in IQtree 2.1.2. ML trees constructed from different filtering criteria produced nearly identical topologies (Fig. S4), therefore we used a data set in which SNPs present in at least 60% of samples (− p 94) for downstream analyses.

We used MrBayes v 3.2^76^ to construct phylogenetic trees using Bayesian inference (BI) with the GTR-I-GAMMA model (General Time Reversible model with a proportion of invariable sites and a gamma-shaped distribution of rates across sites), which includes a parameter for site heterogeneity. BI employed four simultaneous Monte Carlo Markov chains (one cold and three heated) with 1,000,000 generations and sampled every 500 generations. The first 25% of the data points were discarded as burn-in. We confirmed that both ML and Bayesian tree searches achieved convergence (ML tree: Bootstrap correlation coefficient of split occurrence frequencies = 0.99, Bayesian tree: PSRFs = 1.000592). The consensus trees from both ML and BI were illustrated using FigTree v 1.4.3 (http://tree.bio.ed.ac.uk/software/Figtree/).

### Species tree and species delimitation

Species tree inference and species delimitation in a Bayesian framework using SNP data were investigated using SNAPP^77^ and MODEL_SELECTION implemented in BEAST version 2^78^. Species Trees were inferred directly from SNP markers by bypassing gene trees in a full coalescent analysis, using a polynomial-time algorithm that computes the likelihood of a species tree directly from the markers under a finite-sites model of mutation effectively integrating over all possible gene trees^77^. Out of 148 samples for *Cyprogenia*, a total of 40 samples for ten sampling sites (four samples per each sampling site) were aligned and selected based on two sampling criteria, random sampling of individuals or by selecting the individual samples with the highest coverage (smallest number of missing SNPs) using a custom Python script. Coalescent-based species tree was inferred using XML file with following model parameters; with selecting “Cal mutation rates”, unselecting "Include non-polymorphic sites" and selecting "Use Log Likelihood Correction" in Model Parameter, and the chain length of 5,000,000 with storing every 1000 in MCMC, and the rest of parameters were set to default.

For species delimitation exploration, candidate species delimitation models were explored using marginal likelihood estimation (MLE) of each competing species delimitation model, ranking models by marginal likelihood, and using Bayes factors^79^ to assess support for model rankings. For species delimitation using genetic data and coalescent methods, we designed six candidate species delimitation models; Model 1: current taxonomy (two *Cyprogenia* species), Model 2: split *C. aberti* based on highland regions into two species (*C. aberti* was split into Ozark (Black, St. Francis, Spring) and Ouachita (Caddo, Ouachita, Saline)), Model 3: intermix half of two candidate *C. aberti* species from each other (Half of Ozark and Ouachita were equally intermixed), Model 4: reassign *C. aberti* (Black from Ozark was moved to Ouachita, and Saline from Ouachita was moved to Ozark), Model 5: reassign *C. aberti* (Saline from Ouachita was moved to Ozark), Model 6: reassign *C. aberti* (Black from Ozark was moved to Ouachita). For current and alternative species delimitation models, we edited the XML file for marginal likelihood estimation, with the path sampling steps of 36, MCMC sample length of 100,000 for each path sampling step, alpha of 0.3, burnInPercentage = 10, and preBurnin of 10,000. Our path sampling parameters might be low, and a thorough analysis may require more path sampling steps and higher MCMC. However, given computational time and capacity, and similar results from various parameter sets applied, such parameter sets are reasonable.

Bayes factor (BF) calculations based on marginal likelihood estimation (MLE) of each competing species delimitation model are made against the current taxonomy model (Model 1), subtracting the MLE values for two models, and then multiplying the difference by two (BF = 2 × (Model 1 − alternative model). The strength of support from BF comparisons of competing models was evaluated using the framework of Kass and Raftery^79^. The BF scale is as follows: 0 < BF < 2 is not worth more than a bare mention, 2 < BF < 6 is positive evidence, 6 < BF < 10 is strong support, and BF > 10 is decisive. All Bayes factor (BF) calculations are made against the current taxonomy model (Model 1). Therefore, positive BF values indicate support for current taxonomy model, and negative BF values indicate support for alternative model.

Trace files generated by SNAPP were inspected and convergence of chain by checking Effective Sample Size (ESS) for various variables was evaluated using the program Tracer (http://tree.bio.ed.ac.uk/software/tracer). Posterior distribution of species trees was summarized to identify the topology with the best posterior support using TreeAnnotator implemented in BEAST version 2^78^. The target tree file generated with Maximum clade credibility (MCC) and Median heights option in TreeAnnotator was displayed in FigTree.

### Demographic changes in *Cyprogenia*

We investigated the demographic changes for each of the *Cyprogenia* sampling locations in the three highland regions using the Approximate Bayesian Computation (ABC) approach implemented in DIYABC 2.1^80^. To evaluate the past demographic changes, we applied the methods of Cabrera and Palsbøll^81^. Five demographic models were evaluated. Model 1: CON (constant population size), Model 2: DEC (a single instantaneous decrease in population size), Model 3: INC (a single instantaneous increase in population size), Model 4: INCDEC (a single instantaneous increase followed by a single instantaneous decrease in population size), Model 5: DECINC (a single instantaneous decrease followed by a single instantaneous increase in population size). These five models were tested for each of sampling locations and the best fit model was selected by the model comparison implemented in the DIYABC 2.1. We followed the prior model parameterization designed by Cabrera and Palsbøll^81^ with the slight modification of generation time (T) and a uniform distribution with a range of 10–20,000 (N). Similar to Cabrera and Palsbøll^81^, the Last Glacial Maximum (LGM) of approximately 26,500–15,000 years ago was employed as the main cause for changes in population size of these species. In the case of the models DEC and INC, the change in population size occurred after the LGM (i.e., approximately 11,250 years ago), and in the case of the models INCDEC and DECINC, the changes in population size occurred just before and just after the LGM (i.e., approximately 22,500 and 11,250 years ago, respectively)^81^. We employed a generation time of 5 years for *Cyprogenia*, therefore a uniform prior distribution with a range of 100–2250 for T1 (after the LGM), and a range of 2250–4500 for T2 (before the LGM). These prior distributions are reasonable given the species’ presumed census size (100–20,000 mussels) and generation time (5–9 years per generation)^82^.

To minimize effect of missing data in the analyses, we used only loci that were found in all populations using independent parameters of populations program in STACKS for each of three Highlands (-p 3 for 3 populations in Ozark Highland, and − p 3 for 3 populations in Ouachita Highland, and − p 4 for four populations in Eastern Highland). A vcf dataset generated from the Stacks populations command was changed to .snp file using phyton script (vcf2diyabc.py) implemented in the DIYABC program and monomorphic loci were further removed from the dataset so that only polymorphic loci were employed in the ABC analyses. No mutation model parameterization was required for SNPs. Models for demographic changes and their prior distributions were provided in the “Supplementary Information”.

### Genetic relationship among specimens identified by conglutinate lure color

The color of conglutinate lures (red and brown colored eggs) of included specimens were identified previously^23^, and a total of 31 *C. aberti* specimens with known conglutinate lure color (14 with red conglutinates and 17 with brown conglutinates) were selected to examine genetic relationships between specimens. Concatenated SNPs obtained from a moderate criterion (− p 94) described earlier were used for phylogenetic analyses. Maximum likelihood (ML) and Bayesian trees were constructed to examine phylogenetic relationships for total of 33 specimens, 31 *C. aberti* specimens, and two *C. stegeria* specimens used as outgroups. The TVM + I + G4 model of sequence evolution was selected according to a Bayesian Information Criterion (BIC) implemented in IQtree.

### Quality comparison between genetic materials using non-destructive and destructive methods

To investigate the utility of non-destructive methods as source material for genomic library creation, we compared the results obtained from samples collected using cytology brushes and mantle-tissue biopsies. First, we compared the retention rate of samples from each method after the aforementioned filtering steps. Then, we compared the quality and amount of ddRAD sequence reads between genetic samples using both methods. In addition, we estimated and compared differences in SNP error rates between replicated samples from each sample type. Lastly, we mapped and compared ddRAD fastq sequences from all samples to four genome sequences (bivalve, human, yeast and a bacterium) and PhiX sequences using FastQ_Screen (https://www.bioinformatics.babraham.ac.uk/projects/fastq_screen/). Genome sequences of a bivalve, *Mytilus galloprovincialis*, are available from GenBank (accession: LNJA000000000). Genome sequences of human (*Homo sapiens*), and yeast (*Saccharomyces_cerevisiae*) are available from ftp://ftp.ensembl.org/pub/current_fasta. Genome sequences of a bacterium (*Escherichia coli*) are available from GenBank (accession: U00096.3). PhiX sequences are available from Refseq accession NC_001422.1.

## Supplementary Information

Supplementary Information.

## Data Availability

Raw ddRAD-seq data for each sample are deposited in the NCBI SRA database (BioProject ID: PRJNA454895) under the individual SRA accession numbers listed in Table S1. Raw ddRAD-seq data are available in the same BioProject under the SRA Accession no. (SAMN09060972–SAMN09061127).
