# Supplementary material for: Genome-wide SNPs redefines species boundaries and conservation units in the freshwater mussel genus Cyprogenia of North America
Source: Sci Rep. 2021 May 24;11:10752. doi: 10.1038/s41598-021-90325-0 (PMC8144384; doi:10.1038/s41598-021-90325-0)
Supplement: Supplementary file 1 — Supplementary Information. [file 41598_2021_90325_MOESM1_ESM.docx]

**Supplemental Information for:**

Genome-wide SNPs redefines species boundaries and conservation units in the freshwater mussel genus *Cyprogenia* of North America

**Kyung Seok Kim and Kevin J. Roe***

Iowa State University, Department of Natural Resource Ecology and Management, Ames, IA, 50011, USA

Running Title: Fine-scale population structure in *Cyprogenia*

* Corresponding author:

Kevin J. Roe

Iowa State University, Department of Natural Resource Ecology and Management, Ames, IA, 50011, USA

E-mail: kjroe@iastate.edu

**INDEX**

1. **ddRAD loci and quality/sample filtering**
2. **STACKS parameter selection and SNP discovery**
3. **Exploration of demographic history in *Cyprogenia***
4. **References**
5. **Supplementary Tables**
6. **Supplementary Figures**

**I. ddRAD loci and quality/sample filtering**

Process_radtags in the STACKS pipeline (Catchen et al. 2011; 2013) was used to retain the equal size of 85 bp among sequence reads after barcode removal (-t 85), to clean data by removing any sequence reads with an uncalled base (-c), and with low quality score (-q) and to rescue barcodes and restriction enzyme cut sites (-r). NGS sequencing for 238 mussel specimens generated a total of 1,822,173,521 sequence reads for 5 lanes (364,434,704 reads/lane), with an average of 6,207,131 reads per tagged individual after demultiplexing. Of these, 45.2% (average of 2,806,551 per individual) were retained after discarding any reads with low quality scores, uncalled base, ambiguous barcodes and ambiguous rad_tags. There were no major differencesin number of ddRAD raw sequence reads obtained between different indexes within each lane and between different lanes (Fig. S2). We performed several additional quality filtering approaches. Initially, we filtered out 9 of 238 individuals by removing individuals that produced below 25% of the average sequence reads per individual (2,806,551) and with low quality sequence reads using options implemented in STACKS Process_radtags. We constructed concatenated sequences of SNPs recovered from loci that were present in >70% of remaining samples (i.e. 160 of 229) with the population parameter (--phylip_var) and removed an additional 57 individuals that had more than 40% of alleles coded as “N” among those concatenated sequences. Lastly, among replicate samples, the replicate with a lower proportion of “N” was selected for further analyses. In summary, a total of 66 out of the 238 samples neither passed the criterion of 25% of mean number of sequence reads retained per sample, nor the criterion of 40% of ambiguous nucleotide in concatenated SNP sequences (>525 for 1,312 SNPs at -p 160 for 229 samples). After removing replicate samples, a total of 156 samples (148 *Cyprogenia* + 8 *Dromus*) were retained.

**II. STACKS parameter selection and SNP discovery**

A wrapper program (denovo_map.pl) was used to explore the effect of combination of different core parameters (-m, -M, -n) for de novo assembly within STACKS on number of RAD and SNP loci recovered and SNP error rates. A total of 8 replicates (4 replicates from *C. aberti* and4 replicates from *C. stegaria*), that were sequenced from two independent libraries were used to run the wrapper program multiple times with a range of parameter combinations (-m: 2-10, -M: 2-10, -n: 0-7, --max_locus_stacks: 2-6). For each run, only one parameter was varied, with the remaining set to m = 3, M = 2, n = 0 as a default. The maximum number of stacks at a single de novo locus (--max_locus_stacks) was set to 3 (default) to control for confounding loci that may arise from short, sequencing error-based stacks or from repetitive sequences ^1^. We further explored the SNP calling model, by comparing the default SNP model (where error rate varies freely) and the bounded model, testing different values (0.05, 0.1, 0.2, and 0.3) for the upper bound (sequencing error upper bound). Lastly, we applied corrections to genotype and haplotype callsusing STACKS RX program (rxstacks). Outputs were analyzed to estimate the number of RAD loci and SNPs recovered, and SNP error rates using R scripts written by Mastretta_Yanes *et al*.^1^, and to explore the clustering pattern of replicate pairs, the percentage missing data and the proportion of variance using principal component analysis (PCA) (adegenet package in R) using R v. 3.3.3 ^2^.

Once the parameter set with the best performance was selected, we manually ran the STACKS programs, i.e. ustacks, cstacks, sstacks, rxstacks. Specifically, the program cstacks was used to build catalog loci, a set of consensus loci, from 23 samples (20 *Cyprogenia* + 3 *Dromus* samples) that contain the highest number of loci and represent each sampling location and species. Subsequently, STACKS RX program (rxstacks) was used to make corrections to genotype and haplotype calls in individual samples based on population wide data using the following parameters: default SNP model, confounded loci filtering (--conf_lim 0.25), haplotype pruning (--prune_haplo) and minimum log likelihood required to keep a catalog locus (--lnl_lim -10.0). Loci with alleles with minor frequency of less than 0.05 (--min_maf 0.05) were filtered out and a data subset consisting of only the first SNP per ddRAD-Seq locus (--write_sinlge_snp) were retained to minimize effects of linkage disequilibrium.

Across all explored parameter profiles, the number of loci retained ranged from 46,544 to 491,466 and the number of SNPs ranged from 20,386 to 113,024 (Fig. S2A). In general, three main parameter components that control the minimal coverage (-m), and number of mismatches allowed between loci when processing a single individual (-M) and building the catalogue of loci (-n) greatly contributed to the variance of the number of loci retained and SNPs. As the mean coverage per locus increase (the min. coverage -m from 2 to 10), both the number of loci retained and SNPs substantially decreased (Fig. S2B), whereas increasing the number of mismatches allowed between loci when building the catalogue of loci (-n from 0 to 7), produced decreased number of loci retained but increased SNPs (Fig. S2B). Increasing the number of mismatches allowed between loci when processing a single individual (-M) did not affect the number of loci retained but increased the number of SNPs. On the other hand, a parameter that controls the maximum number of stacks at a single de novo locus (-max_locus_stacks), minimally contributed to the amount of data among different parameter settings.

Various parameters generated different levels of the SNP error rate, ranging from 5.1% to 29.9% depending on replicate samples (Fig. S3). SNP errors between replicates can be caused by allelic dropout due to low coverage, or by the allowance of error-based variation due to PCR and/or sequencing errors during de novo assembly. Indeed, SNP error rates substantially increased as minimum number of reads required to create a stack (-m) and number of mismatches allowed between loci when processing a single individual (-M) increase (Fig. S3A, B). The lowest mean SNP error rates (8.6%) was detected in a parameter set of -m 3, -M 2, and -n 3 (Fig. S3C). Again, a parameter, -max_locus_stacks (2-6), did not affect the SNP error rates (Fig. S3D). We further explored SNP calling models with the best performance among replicates using a selected parameter set (-m 3, -M 2, -n 3). SNP calling models did not affect the number of loci retained. However, increasing the value of the upper bounded model (from 0.05 to 0.3) revealed slight decrease in number of SNP loci, with the lowest number of SNP loci in the default SNP model. On the other hand, mean SNP error rate was the lowest in the default SNP model (8.6%), but highest in the parameter --bound_high 0.05 (11.3%). Application of correction program (rxstacks) and the selection of the first SNP per locus (--write_single_snp) further improved SNP error rates from 5.7% (without correction) to 5.2% (with correction) (Fig. S3E), but traded off the recovery of some SNP loci (from 31,945 to 25,806). PCA clustering with correction resulted in increased power in Proportion of Variance in first two components (from 39.5% without correction to 44.2% with correction) as well as in proportion of missing data (from 60.62 % without correction to 54.34 % with correction). In general, PCA analysis showed that most replicates were clustered together regardless of parameter settings, but corrected data resulted in minimizing differences between replicates and increasing discrimination between species (data not shown).

Therefore, results from various exploratory analyses in STACKS revealed that assembly parameters of -m = 3, -M = 2, -n = 3, --max_locus_stacks = 3, and default SNP model with application of correction program (rxstacks) and selection of the first SNP per locusperformed better than other parameter settings and therefore, this parameter setting was chosen for de novo RADseq assembly and SNP discovery, and for downstream genetic analyses.

**III. Exploration of demographic history in *Cyprogenia***

Demographic history of each of the sampling locations for each highland region was explored using the Approximate Bayesian Computation (ABC) approach implemented in DIYABC 2.1 ^3^.

To evaluate the past demographic changes, we followed the methodology employed by Cabrera and Palsbøll ^4^. Five demographic models were evaluated. Model 1: CON (constant population size), Model 2: DEC (a single instantaneous decrease in population size), Model 3: INC (a single instantaneous increase in population size), Model 4: INCDEC (a single instantaneous increase followed by a single instantaneous decrease in population size), Model 5: DECINC (a single instantaneous decrease followed by a single instantaneous increase in population size). These five models were tested for each sampling location and the best fit model was selected by the model comparison implemented in the DIYABC 2.1. We followed the prior model parameterization designed by Cabrera and Palsbøll ^4^ with the slight modification of generation time (T) and a uniform distribution with a range of 10 to 20,000 (N). As in Cabrera and Palsbøll ^4^, the Last Glacial Maximum (LGM) of approximately 26,500 to 15,000 years ago was employed as the main cause for changes in population of the species. In the case of the models DEC and INC, the change in population size occurred after the LGM (i.e., approximately 11,250 years ago), and in the case of the models INCDEC and DECINC, the changes in population size occurred just before and after the LGM (i.e., approximately 22,500 and 11,250 years ago, respectively) ^4^. We used a generation time of 5 years for *Cyprogenia*, therefore a uniform prior distribution with a range of 100 to 2,250 for T1 (after the LGM), and a range of 2,250 to 4,500 for T2 (before the LGM). These prior distributions are reasonable given the species’ presumed census size (100-20,000 mussels) and generation time (5-9 years per generation) ^5^.

Models for demographic changes (5 scenarios) and their corresponding prior distributions are summarized below.

**Models for demographic changes, evolutionary process scenarios and their prior distributions**

1. **Five demographic models from** Cabrera and Palsbøll ^4^

scenario 1: CON (constant population size)

N

0 sample 1

scenario 2: DEC (a single instantaneous decrease in population size)

N

0 sample 1

TT varNe 1 N1b

scenario 3: INC (a single instantaneous increase in population size)

N

0 sample 1

TT varNe 1 N1s

scenario 4: INCDEC (a single instantaneous increase followed by a single instantaneous decrease in population size)

N

0 sample 1

T1 varNe 1 N1bA

T2 varNe 1 N2sA

scenario 5: DECINC (a single instantaneous decrease followed by a single instantaneous increase in population size)

N

0 sample 1

T1 varNe 1 N1sB

T2 varNe 1 N2bB

historical parameters priors

N N UN[10,20000,0.0,0.0]

TT T UN[100,4500,0.0,0.0]

N1b N UN[10,20000,0.0,0.0]

N1s N UN[10,20000,0.0,0.0]

T1 T UN[100,2250,0.0,0.0]

N1bA N UN[10,20000,0.0,0.0]

T2 T UN[2250,4500,0.0,0.0]

N2sA N UN[10,20000,0.0,0.0]

N1sB N UN[10,20000,0.0,0.0]

N2bB N UN[10,20000,0.0,0.0]

T1<T2

N1b>N

N1s<N

N1bA>N

N1bA>N2sA

N1sB<N

N1sB<N2bB

DRAW UNTIL

**IV. References**

1. Mastretta-Yanes, A. *et al.* Restriction site-associated DNA sequencing, genotyping error estimation and de novo assembly optimization for population genetic inference. *Mol. Ecol. Resour.* **15**, 28–41 (2015).

2. R Core Team. R: A Language and Environment for Statistical Computing. R Foundation for Statistical Computing. (2012).

3. Cornuet, J.-M. *et al.* DIYABC v2.0: a software to make approximate Bayesian computation inferences about population history using single nucleotide polymorphism, DNA sequence and microsatellite data. *Bioinformatics* **30**, 1187–1189 (2014).

4. Cabrera, A. A. & Palsbøll, P. J. Inferring past demographic changes from contemporary genetic data: A simulation-based evaluation of the ABC methods implemented in diyabc. *Mol. Ecol. Resour.* **17**, e94–e110 (2017).

5. Jones, J. W. . & Neves, R. J. . Life history and propagation of the endangered fanshell pearlymussel , Cyprogenia stegaria Rafinesque ( Bivalvia : Unionidae ) Author ( s ): Jess W . Jones and Richard J . Neves Published by : The University of Chicago Press on behalf of the Society for F. *J. North Am. Benthol. Soc.* **21**, 76–88 (2002).

**V. Supplementary Tables**

**Table S1** Sample information of freshwater mussels (*Cyprogenia sp.* and *Dromus sp.)* used in this study

| Species | Sample ID | Tree OTU | River, State | Sampling method | Main Clade (subclade) | Genetic cluster -subcluster | Conglutinate ID (Color) | NCBI_SRA | Reference/Collector |
| --- | --- | --- | --- | --- | --- | --- | --- | --- | --- |
| *Cyprogenia stegaria* | CST_01 | Eastern1 | Salt River, KY | Mantle | A | I-1 |  | SAMN09060972 | Grobler et al., 2011 |
| *Cyprogenia stegaria* | CST_02 | Eastern2 | Salt River, KY | Mantle | A | I-1 |  | SAMN09060973 | Grobler et al., 2011 |
| *Cyprogenia stegaria* | CST_03 | Eastern3 | Salt River, KY | Mantle | A | I-1 |  | SAMN09060974 | Grobler et al., 2011 |
| *Cyprogenia stegaria* | CST_04 | Eastern4 | Salt River, KY | Mantle | A | I-1 |  | SAMN09060975 | Grobler et al., 2011 |
| *Cyprogenia stegaria* | CST_05 | Eastern5 | Salt River, KY | Mantle | A | I-1 |  | SAMN09060976 | Grobler et al., 2011 |
| *Cyprogenia stegaria* | CST_06 | Eastern6 | Salt River, KY | Mantle | A | I-1 |  | SAMN09060977 | Grobler et al., 2011 |
| *Cyprogenia stegaria* | CST_07 | Eastern7 | Salt River, KY | Mantle | A | I-1 |  | SAMN09060978 | Grobler et al., 2011 |
| *Cyprogenia stegaria* | CST_08 | Eastern8 | Salt River, KY | Mantle | A | I-1 |  | SAMN09060979 | Grobler et al., 2011 |
| *Cyprogenia stegaria* | CST_09 | Eastern9 | Salt River, KY | Mantle | A | I-1 |  | SAMN09060980 | Grobler et al., 2011 |
| *Cyprogenia stegaria* | UAUC1499 | Eastern11 | Clinch River, TN | Mantle | A | I-1 |  | SAMN09060981 | Serb, 2006 |
| *Cyprogenia stegaria* | UAUC1500 | Eastern12 | Clinch River, TN | Mantle | A | I-1 |  | SAMN09060982 | Serb, 2006 |
| *Cyprogenia stegaria* | CCB_01 | Eastern13 | Clinch River, TN | Mantle | A | I-1 |  | SAMN09060983 | Grobler et al., 2011 |
| *Cyprogenia stegaria* | CCB_02 | Eastern14 | Clinch River, TN | Mantle | A | I-1 |  | SAMN09060984 | Grobler et al., 2011 |
| *Cyprogenia stegaria* | CCF_01 | Eastern15 | Clinch River, TN | Mantle | A (2) | I-2 |  | SAMN09060985 | Grobler et al., 2011 |
| *Cyprogenia stegaria* | CCF_03 | Eastern17 | Clinch River, TN | Mantle | A | I-1 |  | SAMN09060986 | Grobler et al., 2011 |
| *Cyprogenia stegaria* | CCF_04 | Eastern18 | Clinch River, TN | Mantle | A (2) | I-2 |  | SAMN09060987 | Grobler et al., 2011 |
| *Cyprogenia stegaria* | CCF_05 | Eastern19 | Clinch River, TN | Mantle | A | I-1 |  | SAMN09060988 | Grobler et al., 2011 |
| *Cyprogenia stegaria* | CGE_01 | Eastern21 | Green River, KY | Mantle | A | I-1 |  | SAMN09060989 | Grobler et al., 2011 |
| *Cyprogenia stegaria* | CGE_02 | Eastern22 | Green River, KY | Mantle | A | I-1 |  | SAMN09060990 | Grobler et al., 2011 |
| *Cyprogenia stegaria* | CGE_03 | Eastern23 | Green River, KY | Mantle | A | I-1 |  | SAMN09060991 | Grobler et al., 2011 |
| *Cyprogenia stegaria* | CGE_04 | Eastern24 | Green River, KY | Mantle | A | I-1 |  | SAMN09060992 | Grobler et al., 2011 |
| *Cyprogenia stegaria* | CGE_05 | Eastern25 | Green River, KY | Mantle | A | I-1 |  | SAMN09060993 | Grobler et al., 2011 |
| *Cyprogenia stegaria* | CGE_06 | Eastern26 | Green River, KY | Mantle | A | I-1 |  | SAMN09060994 | Grobler et al., 2011 |
| *Cyprogenia stegaria* | CGE_07 | Eastern27 | Green River, KY | Mantle | A | I-1 |  | SAMN09060995 | Grobler et al., 2011 |
| *Cyprogenia stegaria* | CGE_08 | Eastern28 | Green River, KY | Mantle | A | I-1 |  | SAMN09060996 | Grobler et al., 2011 |
| *Cyprogenia stegaria* | CGE_09 | Eastern29 | Green River, KY | Mantle | A | I-1 |  | SAMN09060997 | Grobler et al., 2011 |
| *Cyprogenia stegaria* | CGE_10 | Eastern30 | Green River, KY | Mantle | A | I-1 |  | SAMN09060998 | Grobler et al., 2011 |
| *Cyprogenia stegaria* | CLR_01 | Eastern31 | Licking River, KY | cytology brushes | A | I-1 |  | SAMN09060999 | Chong et al., 2016 |
| *Cyprogenia stegaria* | CLR_02 | Eastern32 | Licking River, KY | cytology brushes | A | I-1 |  | SAMN09061000 | Chong et al., 2016 |
| *Cyprogenia stegaria* | CLR_03 | Eastern33 | Licking River, KY | cytology brushes | A | I-1 |  | SAMN09061001 | Chong et al., 2016 |
| *Cyprogenia stegaria* | CLR_04 | Eastern34 | Licking River, KY | cytology brushes | A | I-1 |  | SAMN09061002 | Chong et al., 2016 |
| *Cyprogenia stegaria* | CLR_05 | Eastern35 | Licking River, KY | cytology brushes | A | I-1 |  | SAMN09061003 | Chong et al., 2016 |
| *Cyprogenia stegaria* | CLR_06 | Eastern36 | Licking River, KY | cytology brushes | A | I-1 |  | SAMN09061004 | Chong et al., 2016 |
| *Cyprogenia stegaria* | CLR_07 | Eastern37 | Licking River, KY | cytology brushes | A | I-1 |  | SAMN09061005 | Chong et al., 2016 |
| *Cyprogenia stegaria* | CLR_09 | Eastern39 | Licking River, KY | cytology brushes | A | I-1 |  | SAMN09061006 | Chong et al., 2016 |
| *Cyprogenia stegaria* | CLR_10 | Eastern40 | Licking River, KY | cytology brushes | A | I-1 |  | SAMN09061007 | Chong et al., 2016 |
| *Cyprogenia stegaria* | CLR_11 | Eastern41 | Licking River, KY | cytology brushes | A | I-1 |  | SAMN09061008 | Chong et al., 2016 |
| *Cyprogenia stegaria* | CLR_12 | Eastern42 | Licking River, KY | cytology brushes | A | I-1 |  | SAMN09061009 | Chong et al., 2016 |
| *Cyprogenia stegaria* | CLR_13 | Eastern43 | Licking River, KY | cytology brushes | A | I-1 |  | SAMN09061010 | Chong et al., 2016 |
| *Cyprogenia stegaria* | CLR_14 | Eastern44 | Licking River, KY | cytology brushes | A | I-1 |  | SAMN09061011 | Chong et al., 2016 |
| *Cyprogenia stegaria* | CLR_15 | Eastern45 | Licking River, KY | cytology brushes | A | I-1 |  | SAMN09061012 | Chong et al., 2016 |
| *Cyprogenia stegaria* | CLR_17 | Eastern46 | Licking River, KY | cytology brushes | A | I-1 |  | SAMN09061013 | Chong et al., 2016 |
| *Cyprogenia stegaria* | CLR_18 | Eastern47 | Licking River, KY | cytology brushes | A | I-1 |  | SAMN09061014 | Chong et al., 2016 |
| *Cyprogenia stegaria* | CLR_19 | Eastern48 | Licking River, KY | cytology brushes | A | I-1 |  | SAMN09061015 | Chong et al., 2016 |
| *Cyprogenia stegaria* | CLR_20 | Eastern49 | Licking River, KY | cytology brushes | A | I-1 |  | SAMN09061016 | Chong et al., 2016 |
| *Cyprogenia stegaria* | CLR_21 | Eastern50 | Licking River, KY | cytology brushes | A | I-1 |  | SAMN09061017 | Chong et al., 2016 |
| *Cyprogenia stegaria* | CLR_22 | Eastern51 | Licking River, KY | cytology brushes | A | I-1 |  | SAMN09061018 | Chong et al., 2016 |
| *Cyprogenia stegaria* | CLR_23 | Eastern52 | Licking River, KY | cytology brushes | A | I-1 |  | SAMN09061019 | Chong et al., 2016 |
| *Cyprogenia stegaria* | CLR_24 | Eastern53 | Licking River, KY | cytology brushes | A | I-1 |  | SAMN09061020 | Chong et al., 2016 |
| *Cyprogenia stegaria* | CLR_25 | Eastern54 | Licking River, KY | cytology brushes | A | I-1 |  | SAMN09061021 | Chong et al., 2016 |
| *Cyprogenia stegaria* | CLRd_16 | Eastern55 | Licking River, KY | cytology brushes | A | I-1 |  | SAMN09061022 | Chong et al., 2016 |
| *Cyprogenia aberti* | COR_02 | Ouachi2 | Ouachita River, AR | Mantle | C-1 | III-1 | R_OuaOC4 (Red) | SAMN09061023 | Chong et al., 2016 |
| *Cyprogenia aberti* | COR_03 | Ouachi3 | Ouachita River, AR | Mantle | C-1 | III-1 | R_OuaOC5 (Red) | SAMN09061024 | Chong et al., 2016 |
| *Cyprogenia aberti* | CSL_01 | Ouachi20 | Saline River, AR | Mantle | C-2 | III-2 | R_SalOC9 (Red) | SAMN09061025 | Chong et al., 2016 |
| *Cyprogenia aberti* | CSL_02 | Ouachi21 | Saline River, AR | Mantle | C-2 | III-2 | B_SalOC12 (Brown) | SAMN09061026 | Chong et al., 2016 |
| *Cyprogenia aberti* | CSR_01 | Ouachi22 | Saline River, AR | Mantle | C-2 | III-2 | B_SalOC13 (Brown) | SAMN09061027 | Chong et al., 2016 |
| *Cyprogenia aberti* | CSR_02 | Ouachi23 | Saline River, AR | Mantle | C-2 | III-2 | B_SalOC14 (Brown) | SAMN09061028 | Chong et al., 2016 |
| *Cyprogenia aberti* | CSR_03 | Ouachi24 | Saline River, AR | Mantle | C-2 | III-2 | B_SalOC15 (Brown) | SAMN09061029 | Chong et al., 2016 |
| *Cyprogenia aberti* | CSR_04 | Ouachi25 | Saline River, AR | Mantle | C-2 | III-2 | B_SalOC16 (Brown) | SAMN09061030 | Chong et al., 2016 |
| *Cyprogenia aberti* | CSR_05 | Ouachi26 | Saline River, AR | Mantle | C-2 | III-2 | B_SalOC17 (Brown) | SAMN09061031 | Chong et al., 2016 |
| *Cyprogenia aberti* | CSR_06 | Ouachi27 | Saline River, AR | Mantle | C-2 | III-2 | R_SalOC10 (Red) | SAMN09061032 | Chong et al., 2016 |
| *Cyprogenia aberti* | CSR_07 | Ouachi28 | Saline River, AR | Mantle | C-2 | III-2 | R_SalOC11 (Red) | SAMN09061033 | Chong et al., 2016 |
| *Cyprogenia aberti* | CSR_08 | Ouachi29 | Saline River, AR | Mantle | C-2 | III-2 | R_SalOC12 (Red) | SAMN09061034 | Chong et al., 2016 |
| *Cyprogenia aberti* | CSR_09 | Ouachi30 | Saline River, AR | Mantle | C-2 | III-2 | R_SalOC13 (Red) | SAMN09061035 | Chong et al., 2016 |
| *Cyprogenia aberti* | CSR_10 | Ouachi31 | Saline River, AR | Mantle | C-2 | III-2 | R_SalOC14 (Red) | SAMN09061036 | Chong et al., 2016 |
| *Cyprogenia aberti* | UAUC1837 | Ouachi33 | Caddo River, AR | Mantle | C-1 | III-1 |  | SAMN09061037 | Serb, 2006 |
| *Cyprogenia aberti* | UAUC1838 | Ouachi34 | Caddo River, AR | Mantle | C-1 | III-1 |  | SAMN09061038 | Serb, 2006 |
| *Cyprogenia aberti* | UAUC1839 | Ouachi35 | Caddo River, AR | Mantle | C-1 | III-1 |  | SAMN09061039 | John Harris |
| *Cyprogenia aberti* | UAUC2374 | Ouachi36 | Ouachita River, AR | Mantle | C-1 | III-1 |  | SAMN09061040 | Serb, 2006 |
| *Cyprogenia aberti* | UAUC2375 | Ouachi37 | Ouachita River, AR | Mantle | C-1 | III-1 |  | SAMN09061041 | Serb, 2006 |
| *Cyprogenia aberti* | UAUC2377 | Ouachi38 | Ouachita River, AR | Mantle | C-1 | III-1 |  | SAMN09061042 | Serb, 2006 |
| *Cyprogenia aberti* | UAUC2378 | Ouachi39 | Ouachita River, AR | Mantle | C-1 | III-1 |  | SAMN09061043 | Serb, 2006 |
| *Cyprogenia aberti* | UAUC2381 | Ouachi40 | Caddo River, AR | Mantle | C-1 | III-1 |  | SAMN09061044 | Serb, 2006 |
| *Cyprogenia aberti* | UAUC2383 | Ouachi41 | Caddo River, AR | Mantle | C-1 | III-1 |  | SAMN09061045 | Serb, 2006 |
| *Cyprogenia aberti* | UAUC2736 | Ouachi42 | Saline River, AR | Mantle | C-2 | III-2 |  | SAMN09061046 | Serb, 2006 |
| *Cyprogenia aberti* | CBR_07 | Ozark5 | Black River, MO | cytology brushes | B-1 | 1II-1 | R_BlaOZ1 (Red) | SAMN09061047 | Chong et al., 2016 |
| *Cyprogenia aberti* | CBR_09 | Ozark7 | Black River, MO | cytology brushes | B-1 | II-1 | R_BlaOZ2 (Red) | SAMN09061048 | Chong et al., 2016 |
| *Cyprogenia aberti* | CBR_10 | Ozark8 | Black River, MO | Mantle | B-1 | II-1 | R_BlaOZ3 (Red) | SAMN09061049 | Chong et al., 2016 |
| *Cyprogenia aberti* | CBR_13 | Ozark11 | Black River, MO | cytology brushes | B-1 | II-1 | B_BlaOZ1 (Brown) | SAMN09061050 | Chong et al., 2016 |
| *Cyprogenia aberti* | CBR_14 | Ozark12 | Black River, MO | cytology brushes | B-1 | II-1 | B_BlaOZ2 (Brown) | SAMN09061051 | Chong et al., 2016 |
| *Cyprogenia aberti* | CBR_15 | Ozark13 | Black River, MO | Mantle | B-1 | II-1 | B_BlaOZ3 (Brown) | SAMN09061052 | Chong et al., 2016 |
| *Cyprogenia aberti* | CBR_18 | Ozark16 | Black River, MO | Mantle | B-1 | II-1 |  | SAMN09061053 | Chong et al., 2016 |
| *Cyprogenia aberti* | CBR_19 | Ozark17 | Black River, MO | Mantle | B-1 | II-1 |  | SAMN09061054 | Chong et al., 2016 |
| *Cyprogenia aberti* | CBR_27 | Ozark22 | Black River, MO | cytology brushes | B-1 | II-1 |  | SAMN09061055 | Chong et al., 2016 |
| *Cyprogenia aberti* | CBW_02 | Ozark24 | Black River, AR | Mantle | B-1 | II-1 |  | SAMN09061056 | Chong et al., 2016 |
| *Cyprogenia aberti* | CBW_03 | Ozark25 | Black River, AR | Mantle | B-1 | II-1 |  | SAMN09061057 | Chong et al., 2016 |
| *Cyprogenia aberti* | CBW_04 | Ozark26 | Black River, AR | Mantle | B-1 | II-1 |  | SAMN09061058 | Chong et al., 2016 |
| *Cyprogenia aberti* | CBW_05 | Ozark27 | Black River, AR | Mantle | B-1 | II-1 |  | SAMN09061059 | Chong et al., 2016 |
| *Cyprogenia aberti* | CBW_06 | Ozark28 | Black River, AR | Mantle | B-1 | II-1 |  | SAMN09061060 | Chong et al., 2016 |
| *Cyprogenia aberti* | CBW_07 | Ozark29 | Black River, AR | Mantle | B-1 | II-1 |  | SAMN09061061 | Chong et al., 2016 |
| *Cyprogenia aberti* | CBW_08 | Ozark30 | Black River, AR | Mantle | B-1 | II-1 |  | SAMN09061062 | Chong et al., 2016 |
| *Cyprogenia aberti* | CBW_09 | Ozark31 | Black River, AR | Mantle | B-1 | II-1 |  | SAMN09061063 | Chong et al., 2016 |
| *Cyprogenia aberti* | CBW_10 | Ozark32 | Black River, AR | Mantle | B-1 | II-1 |  | SAMN09061064 | Chong et al., 2016 |
| *Cyprogenia aberti* | CBW_11 | Ozark33 | Black River, AR | Mantle | B-1 | II-1 |  | SAMN09061065 | Chong et al., 2016 |
| *Cyprogenia aberti* | CBW_12 | Ozark34 | Black River, AR | Mantle | B-1 | II-1 |  | SAMN09061066 | Chong et al., 2016 |
| *Cyprogenia aberti* | CBW_13 | Ozark35 | Black River, AR | Mantle | B-1 | II-1 |  | SAMN09061067 | Chong et al., 2016 |
| *Cyprogenia aberti* | CBW_14 | Ozark36 | Black River, AR | Mantle | B-1 | II-1 |  | SAMN09061068 | Chong et al., 2016 |
| *Cyprogenia aberti* | CBW_16 | Ozark38 | Black River, AR | Mantle | B-1 | II-1 |  | SAMN09061069 | Chong et al., 2016 |
| *Cyprogenia aberti* | CBW_18 | Ozark40 | Black River, AR | Mantle | B-1 | II-1 |  | SAMN09061070 | Chong et al., 2016 |
| *Cyprogenia aberti* | CBW_19 | Ozark41 | Black River, AR | Mantle | B-1 | II-1 |  | SAMN09061071 | Chong et al., 2016 |
| *Cyprogenia aberti* | CSF_01 | Ozark42 | St. Francis River, MO | Mantle | B-2 | II-2 | R_StFOZ6 (Red) | SAMN09061072 | Chong et al., 2016 |
| *Cyprogenia aberti* | CSF_02 | Ozark43 | St. Francis River, MO | Mantle | B-2 | II-2 | R_StFOZ7 (Red) | SAMN09061073 | Chong et al., 2016 |
| *Cyprogenia aberti* | CSF_03 | Ozark44 | St. Francis River, MO | Mantle | B-2 | II-2 | R_StFOZ8 (Red) | SAMN09061074 | Chong et al., 2016 |
| *Cyprogenia aberti* | CSF_04 | Ozark45 | St. Francis River, MO | Mantle | B-2 | II-2 | B_StFOZ4 (Brown) | SAMN09061075 | Chong et al., 2016 |
| *Cyprogenia aberti* | CSF_05 | Ozark46 | St. Francis River, MO | cytology brushes | B-2 | II-2 | B_StFOZ5 (Brown) | SAMN09061076 | Chong et al., 2016 |
| *Cyprogenia aberti* | CSF_06 | Ozark47 | St. Francis River, MO | cytology brushes | B-2 | II-2 | B_StFOZ6 (Brown) | SAMN09061077 | Chong et al., 2016 |
| *Cyprogenia aberti* | CSF_07 | Ozark48 | St. Francis River, MO | cytology brushes | B-2 | II-2 | B_StFOZ7 (Brown) | SAMN09061078 | Chong et al., 2016 |
| *Cyprogenia aberti* | CSF_08 | Ozark49 | St. Francis River, MO | cytology brushes | B-2 | II-2 | B_StFOZ8 (Brown) | SAMN09061079 | Chong et al., 2016 |
| *Cyprogenia aberti* | CSF_09 | Ozark50 | St. Francis River, MO | cytology brushes | B-2 | II-2 | B_StFOZ9 (Brown) | SAMN09061080 | Chong et al., 2016 |
| *Cyprogenia aberti* | CSF_10 | Ozark51 | St. Francis River, MO | Mantle | B-2 | II-2 | B_StFOZ10 (Brown) | SAMN09061081 | Chong et al., 2016 |
| *Cyprogenia aberti* | CSF_13 | Ozark54 | St. Francis River, MO | cytology brushes | B-2 | II-2 | B_StFOZ11 (Brown) | SAMN09061082 | Chong et al., 2016 |
| *Cyprogenia aberti* | CSF_18 | Ozark59 | St. Francis River, MO | cytology brushes | B-2 | II-2 |  | SAMN09061083 | Chong et al., 2016 |
| *Cyprogenia aberti* | CSF_19 | Ozark60 | St. Francis River, MO | cytology brushes | B-2 | II-2 |  | SAMN09061084 | Chong et al., 2016 |
| *Cyprogenia aberti* | CSF_21 | Ozark62 | St. Francis River, MO | Mantle | B-2 | II-2 |  | SAMN09061085 | Chong et al., 2016 |
| *Cyprogenia aberti* | CSF_24 | Ozark65 | St. Francis River, MO | cytology brushes | B-2 | II-2 |  | SAMN09061086 | Chong et al., 2016 |
| *Cyprogenia aberti* | CSP_01 | Ozark70 | Spring River, AR | Mantle | B-1 | II-1 |  | SAMN09061087 | Chong et al., 2016 |
| *Cyprogenia aberti* | CSP_02 | Ozark71 | Spring River, AR | Mantle | B-1 | II-1 |  | SAMN09061088 | Chong et al., 2016 |
| *Cyprogenia aberti* | CSP_05 | Ozark73 | Spring River, AR | Mantle | B-1 | II-1 |  | SAMN09061089 | Chong et al., 2016 |
| *Cyprogenia aberti* | CSP_06 | Ozark74 | Spring River, AR | Mantle | B-1 | II-1 |  | SAMN09061090 | Chong et al., 2016 |
| *Cyprogenia aberti* | CSP_07 | Ozark75 | Spring River, AR | Mantle | B-1 | II-1 |  | SAMN09061091 | Chong et al., 2016 |
| *Cyprogenia aberti* | CSP_08 | Ozark76 | Spring River, AR | Mantle | B-1 | II-1 |  | SAMN09061092 | Chong et al., 2016 |
| *Cyprogenia aberti* | CSP_09 | Ozark77 | Spring River, AR | Mantle | B-1 | II-1 |  | SAMN09061093 | Chong et al., 2016 |
| *Cyprogenia aberti* | CSP_10 | Ozark78 | Spring River, AR | Mantle | B-1 | II-1 |  | SAMN09061094 | Chong et al., 2016 |
| *Cyprogenia aberti* | CSP_12 | Ozark79 | Spring River, AR | Mantle | B-1 | II-1 |  | SAMN09061095 | Chong et al., 2016 |
| *Cyprogenia aberti* | CSP_13 | Ozark80 | Spring River, AR | Mantle | B-1 | II-1 |  | SAMN09061096 | Chong et al., 2016 |
| *Cyprogenia aberti* | CSP_14 | Ozark81 | Spring River, AR | Mantle | B-1 | II-1 |  | SAMN09061097 | Chong et al., 2016 |
| *Cyprogenia aberti* | CSP_15 | Ozark82 | Spring River, AR | Mantle | B-1 | II-1 |  | SAMN09061098 | Chong et al., 2016 |
| *Cyprogenia aberti* | CSP_16 | Ozark83 | Spring River, AR | Mantle | B-1 | II-1 |  | SAMN09061099 | Chong et al., 2016 |
| *Cyprogenia aberti* | CSP_17 | Ozark84 | Spring River, AR | Mantle | B-1 | II-1 |  | SAMN09061100 | Chong et al., 2016 |
| *Cyprogenia aberti* | CSPd_04 | Ozark86 | Spring River, AR | Mantle | B-1 | II-1 |  | SAMN09061101 | Chong et al., 2016 |
| *Cyprogenia aberti* | CSP_11 | Ozark89 | Spring River, AR | Mantle | B-1 | II-1 |  | SAMN09061102 | Chong et al., 2016 |
| *Cyprogenia aberti* | CSS_01 | Ozark96 | Spring River, AR | Mantle | B-1 | II-1 |  | SAMN09061103 | Chong et al., 2016 |
| *Cyprogenia aberti* | CSS_02 | Ozark97 | Spring River, AR | Mantle | B-1 | II-1 |  | SAMN09061104 | Chong et al., 2016 |
| *Cyprogenia aberti* | CSS_03 | Ozark98 | Spring River, AR | Mantle | B-1 | II-1 |  | SAMN09061105 | Chong et al., 2016 |
| *Cyprogenia aberti* | CSS_04 | Ozark99 | Spring River, AR | Mantle | B-1 | II-1 |  | SAMN09061106 | Chong et al., 2016 |
| *Cyprogenia aberti* | CSS_05 | Ozark100 | Spring River, AR | Mantle | B-1 | II-1 |  | SAMN09061107 | Chong et al., 2016 |
| *Cyprogenia aberti* | CSS_06 | Ozark101 | Spring River, AR | Mantle | B-1 | II-1 |  | SAMN09061108 | Chong et al., 2016 |
| *Cyprogenia aberti* | CSS_07 | Ozark102 | Spring River, AR | Mantle | B-1 | II-1 |  | SAMN09061109 | Chong et al., 2016 |
| *Cyprogenia aberti* | CSS_08 | Ozark103 | Spring River, AR | Mantle | B-1 | II-1 |  | SAMN09061110 | Chong et al., 2016 |
| *Cyprogenia aberti* | UAUC1446 | Ozark104 | St. Francis River. MO | Mantle | B-2 | II-2 |  | SAMN09061111 | Serb, 2006 |
| *Cyprogenia aberti* | UAUC1535 | Ozark105 | Spring River, KS | Mantle | B-3 | II-3 |  | SAMN09061112 | Serb, 2006 |
| *Cyprogenia aberti* | UAUC1536 | Ozark106 | Current, AR | Mantle | B-1 | II-1 |  | SAMN09061113 | Serb, 2006 |
| *Cyprogenia aberti* | UAUC1647 | Ozark107 | Fall River, KS | Mantle | B-3 | II-3 |  | SAMN09061114 | Serb, 2006 |
| *Cyprogenia aberti* | UAUC1650 | Ozark108 | Buffalo, AR | Mantle | B-1 | II-1 |  | SAMN09061115 | Serb, 2006 |
| *Cyprogenia aberti* | UAUC1652 | Ozark109 | Strawberry, AR | Mantle | B-1 | II-1 |  | SAMN09061116 | Serb, 2006 |
| *Cyprogenia aberti* | UAUC2724 | Ozark110 | Black River, AR | Mantle | B-1 | II-1 |  | SAMN09061117 | Serb, 2006 |
| *Cyprogenia aberti* | UAUC2725 | Ozark111 | Black River, AR | Mantle | B-1 | II-1 |  | SAMN09061118 | Serb, 2006 |
| *Cyprogenia aberti* | UAUC2726 | Ozark112 | Strawberry, AR | Mantle | B-1 | II-1 |  | SAMN09061119 | Serb, 2006 |
| *Dromus dromas* | DRO_1R | Dromus1 | Clinch River, TN | cytology brushes | D | IV |  | SAMN09061120 | This study |
| *Dromus dromas* | DRO_2R | Dromus2 | Clinch River, TN | cytology brushes | D | IV |  | SAMN09061121 | This study |
| *Dromus dromas* | DRO_3W | Dromus3 | Clinch River, TN | cytology brushes | D | IV |  | SAMN09061122 | This study |
| *Dromus dromas* | DRO_4U | Dromus4 | Clinch River, TN | cytology brushes | D | IV |  | SAMN09061123 | This study |
| *Dromus dromas* | DRO_6R | Dromus6 | Clinch River, TN | cytology brushes | D | IV |  | SAMN09061124 | This study |
| *Dromus dromas* | DRO_7R | Dromus7 | Clinch River, TN | cytology brushes | D | IV |  | SAMN09061125 | This study |
| *Dromus dromas* | T358 | Dromus11 | Clinch River, TN | cytology brushes | D | IV |  | SAMN09061126 | This study |
| *Dromus dromas* | T360 | Dromus13 | Clinch River, TN | cytology brushes | D | IV |  | SAMN09061127 | This study |

**VI. Supplementary Figures**

**Figure S1.** Flowchart of experimental design from library construction to data analyses in this study. Flowchart shows different datasets (e.g. raw reads, phylogenetic and population genetic RAD loci datasets), data processing steps (e.g. de-multiplexing, loci assembly, loci filtering, and selection of optimal STACKS parameter), genetic analyses (e.g. phylogenetics, population genetic, outlier analyses for conglutinate egg color, and evolutionary model using ABC simulations).


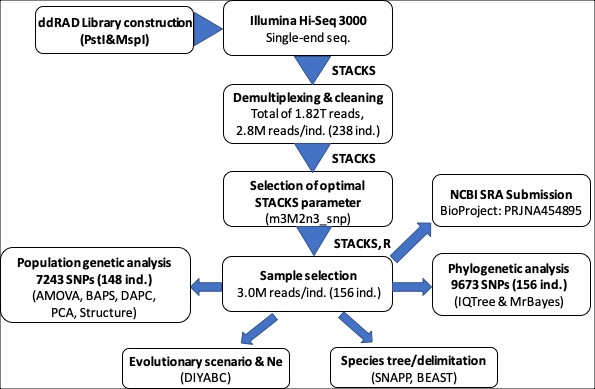
**Figure S2.** Number of ddRAD raw sequence reads and single-nucleotide polymorphisms based on STACKS core parameter settings.

A: Number of ddRAD raw sequence reads obtained between different indexes within each lane and between different lanes

B: Total number of ddRAD loci (Red dotted line) and single-nucleotide polymorphisms (Grey dotted line) obtained using various STACKS core parameter settings. For each run, only one parameter varied, with the remaining set to m = 3, M = 2, n = 0 and max_locus_stacks (--mls) = 3


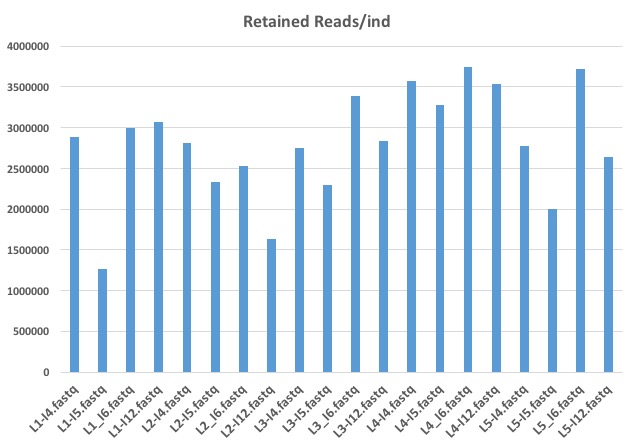


A


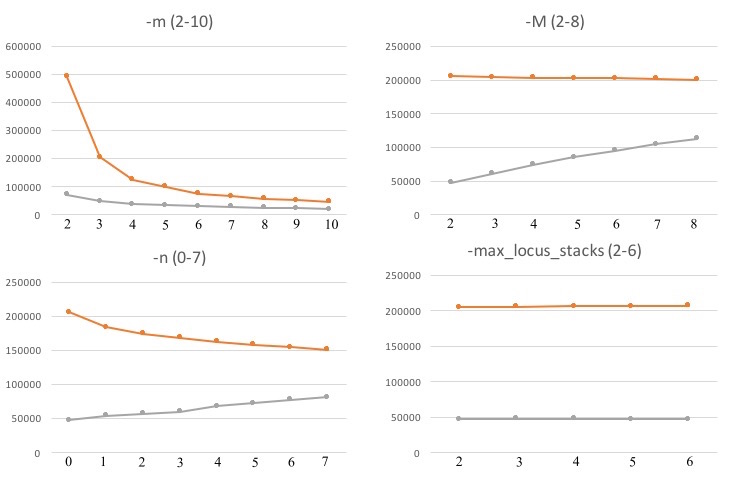


B

**Figure S3.** Effect of different settings for STACKS core parameters and program components on the single nucleotide polymorphism (SNP) error rate.

A: -m = 2-10, B: -M = 2-10, C: -n = 0-7, D: --max_locus_stacks = 2-6 E: SNP calling models (--bound_high 0.05 vs default SNP model), correction program (rxstacks). and the selection of the first SNP per locus(--write_single_snp)

For each run, only one parameter varied (shown on the x axis), with the remaining set to m = 3, M = 2, n = 0 and max_locus_stacks (mx.lcs) = 3


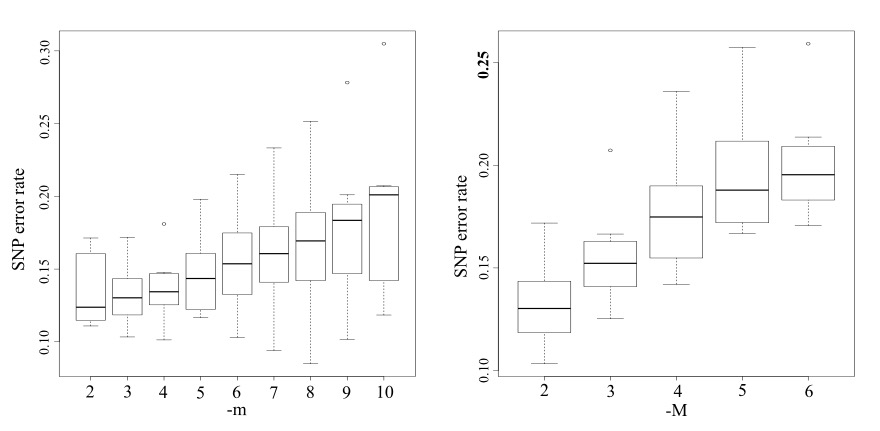


A B


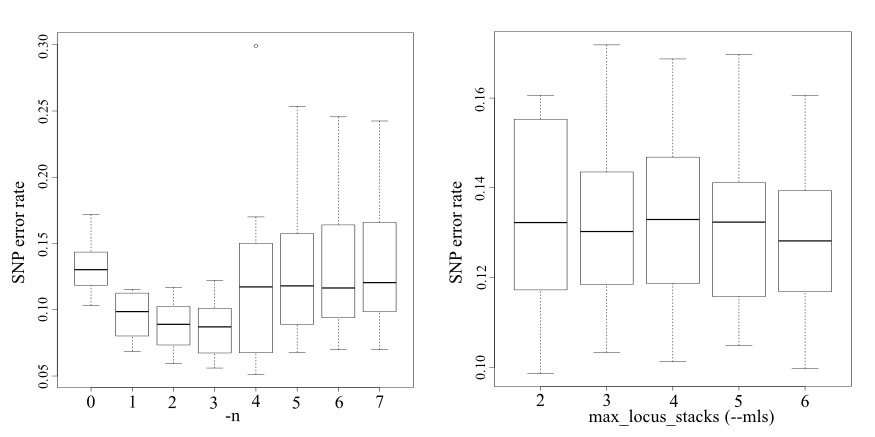
 C D


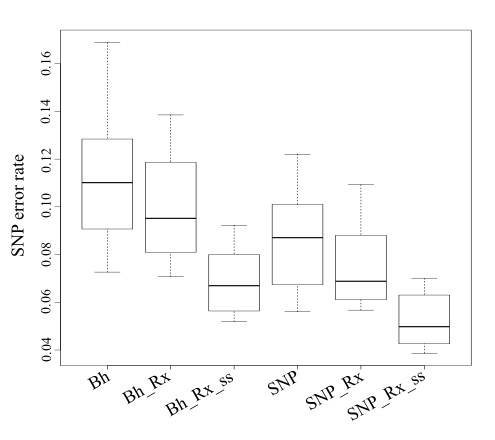


E

**Figure S4.** Phylogenetic relationships of freshwater mussel species based on maximum likelihood (ML) analysis using concatenated SNPs obtained from four different filtering criteria with ascertainment bias correction using the IQTree.

ML trees from different filtering criteria, -p 78, -p 94, -p 109, nd -p 125, where -p refers to the minimum number of samples that a locus must be present in for inclusion of the locus in the phylogenetic analysis. Trees were constructed based on 7,440, 4,395, 2,014, and 511, informative sites respectively. All datasets selected the same TVM+F+ASC+G4 as a best-fit model according to BIC implemented in IQtree. Correspondence of each color to sampling site is represented in Fig. 3.


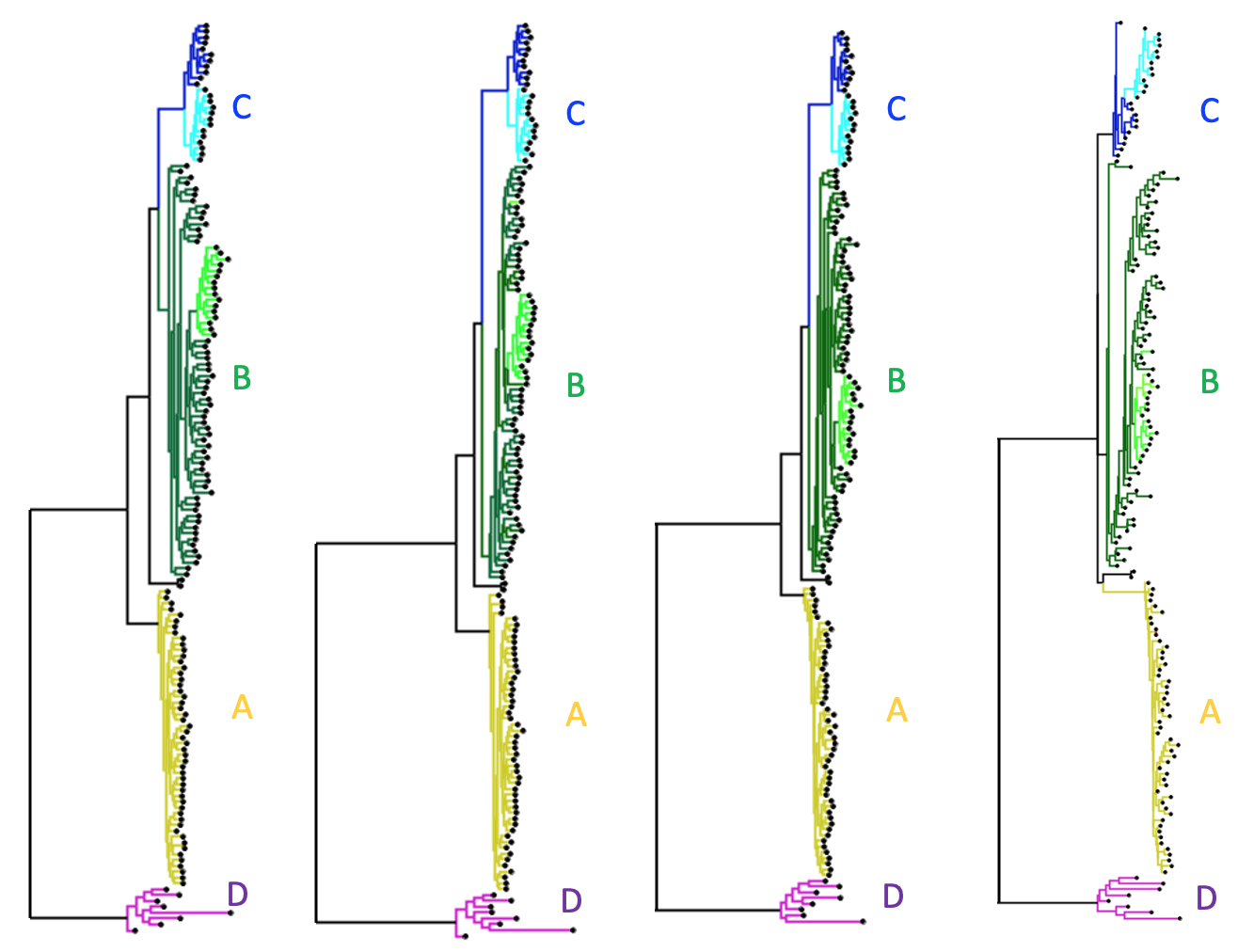


-p 78 -p 94 -p 109 -p 125

**Figure S5.** Phylogenetic trees for 31 *C. aberti* specimens with identified egg colors of conglutinate lure.

Maximum likelihood (ML) tree (A) and Bayesian tree (B) were constructed based on 1,173 informative sites to examine phylogenetic relationships for 33 specimens, including 31 *C. aberti* specimens identified by conglutinate egg colors, and two *C. stegeria* specimens used as an outgroup. The TVM+I+G4 model of sequence evolution was selected following a Bayesian Information Criterion (BIC) implemented in IQtree. Red branch refers to specimens with red color of conglutinate lure and brown branch to specimens with brown color of conglutinate lure)


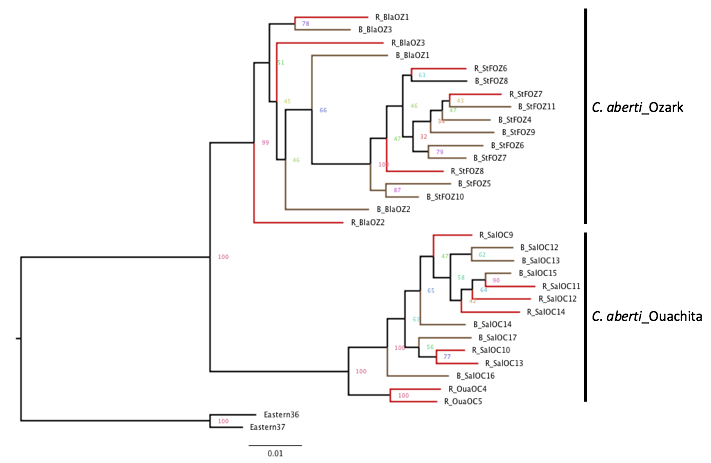


A


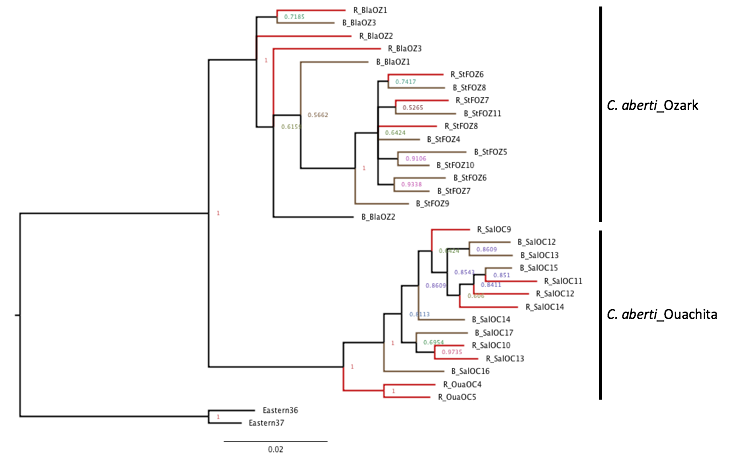


B

**Figure S6.** Comparison of various estimates (Mean±SD) of ddRAD loci from 110 destructive (mantle biopsy) and 46 non-destructive (cytology brush) samples obtained after STACKS process_radtags running.

Number of retained loci (Retained) was obtained after subtraction of loci with no radtag (NoRadtags) and low quality (LowQuality) from total number of ddRAD loci generated.


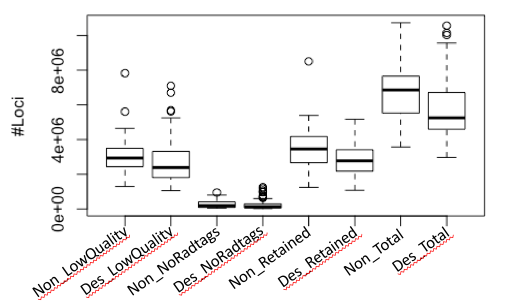


**Figure S7.** Mapping results of destructive and nondestructive genetic samples using FastQ_Screen

ddRAD fastq sequences of two randomly selected were mapped to four genome sequences and PhiX sequences.

A: CSF_21 (Sample using destructive method: 3,023,365 loci)

B: CSF_19 (Sample using non-destructive cytology brush: 3,881,449 loci)

Genome sequences of a bivalve, *Mytilus galloprovincialis*, are available from GenBank (accession: LNJA000000000). Genome sequences of human (*Homo sapiens*), and yeast (*Saccharomyces_cerevisiae*) are available from <ftp://ftp.ensembl.org/pub/current_fasta>. Genome sequences of a bacterium (*Escherichia coli*) are available from GenBank (accession: U00096.3). PhiX sequences are available from Refseq accession NC_001422.1


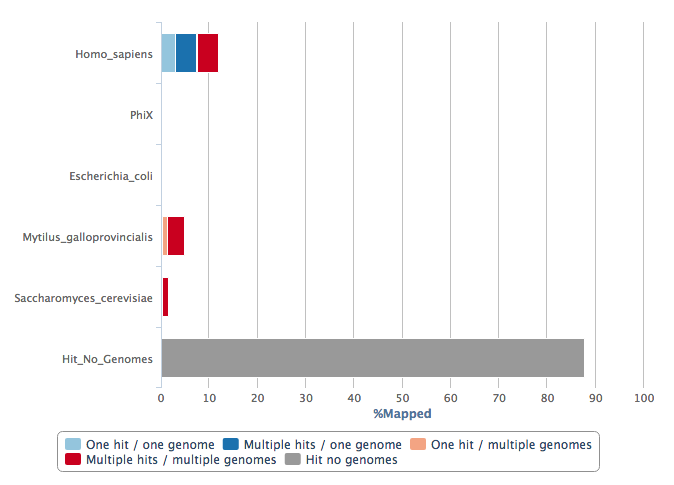


A

**
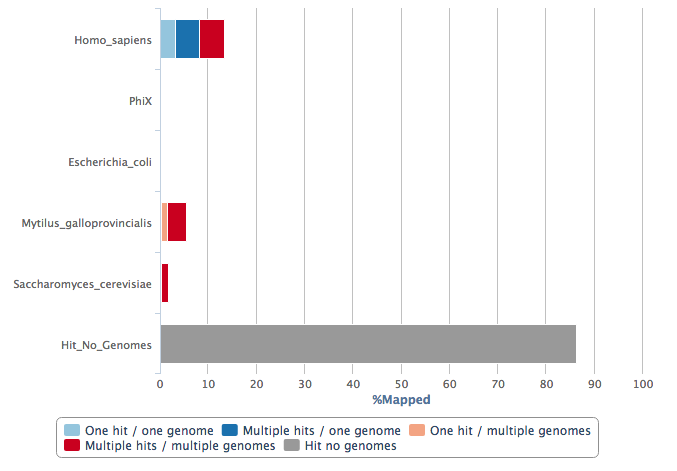
**

B
